# Open-source quality assurance for multi-parametric MRI: a diffusion analysis update for the magnetic resonance biomarker assessment software (MR-BIAS)

**DOI:** 10.1007/s10334-025-01252-4

**Published:** 2025-04-26

**Authors:** James C. Korte, Stanley A. Norris, Madeline E. Carr, Lois Holloway, Glenn D. Cahoon, Ben Neijndorff, Petra van Houdt, Rick Franich

**Affiliations:** 1 Department of Physical Sciences, Peter MacCallum Cancer Centre, 305 Grattan Street, Melbourne, VIC 3000 Australia; 2 Department of Biomedical Engineering, The University of Melbourne, Melbourne, Australia; 3 School of Science, RMIT University, Melbourne, Australia; 4 Monash Imaging, Monash Health, Melbourne, Australia; 5 Centre for Medical Radiation Physics, University of Wollongong, Wollongong, Australia; 6 Liverpool and Macarthur Cancer Therapy Centres and Ingham Institute, Sydney, Australia; 7 GenesisCare, Sydney, NSW Australia; 8 Olivia Newton John Cancer and Wellness Centre, Melbourne, Australia; 9 Department of Radiation Oncology, The Netherlands Cancer Institute, Amsterdam, The Netherlands

**Keywords:** Diffusion magnetic resonance imaging (D038524), Multiparametric magnetic resonance imaging (D000081364), Phantoms, Imaging (D019047), Software validation (D012986), Software [open source software] (D012984)

## Abstract

**Objective:**

To validate the automated analysis of magnetic resonance imaging (MRI) diffusion phantoms with an updated version of the magnetic resonance biomarker assessment software (MR-BIAS), an open-source tool initially developed for the analysis of MRI relaxometry phantoms.

**Materials and methods:**

The updated MR-BIAS was validated against two published diffusion weighted MRI datasets: (i) a single-site study (*n* = 48) was used for validation of apparent diffusion coefficients (ADC) and to identify optimal region of interest (ROI) selection, and (ii) a multi-centre multi-vendor study including diffusion imaging from a shared benchmark protocol (*n* = 49) and site-specific protocols (*n* = 43). ADC analysis compared both datasets with ROIs manually matched to the original studies, and with automatically detected optimal ROIs.

**Results:**

MR-BIAS ADC values were statistically equivalent (*p* < 0.05) to original studies within tolerances (manual ROI, automatic ROI) for the single-site study (± 0.01, ± 6 μm^2^/s) and for the multi-vendor study for benchmark (± 4, ± 7 μm^2^/s) and site-specific (± 3, ± 6 μm^2^/s) protocols. The optimal ROI was a central cylinder (height = 10mm, diamete*r* = 10mm). MR-BIAS ADC summary metrics were comparable to those of the original studies.

**Discussion:**

MR-BIAS can automatically and accurately perform ADC analysis of diffusion phantoms, making the software suitable for the quality assurance of multi-centre studies of multi-parametric MRI.

**Supplementary Information:**

The online version contains supplementary material available at 10.1007/s10334-025-01252-4.

## Introduction

Magnetic resonance imaging (MRI) is a widely used diagnostic imaging modality. Clinical decisions are often guided by radiologists and other medical professionals, based on the qualitative interpretation of relative intensities in MRI images. Quantitative MRI (qMRI) has the potential to numerically identify changes in the patient, such as detecting a region with an apparent diffusion coefficient (ADC) outside of an expected range for healthy tissue of a specific organ. Quantitative measurements require accurate, repeatable, and reproducible values across different MRI scanners. Robust qMRI methods would improve our ability to compare quantitative biomarker values between departments, patients, and across timepoints [1], allowing larger population studies and an improved understanding of longitudinal tissue changes. Additionally, evidence suggests that qMRI biomarkers could facilitate more timely treatment interventions, as they may be able to detect early functional changes in tumours, such as changes in vascularity or cellularity, which often precede later changes in tumour shape or volume [2]. Widespread adoption of qMRI methods has the potential to improve screening, diagnosis, prognosis, and monitoring of response during and after therapy [3].

The clinical implementation of qMRI biomarkers requires technical validation [4–7] and an understanding of measurement uncertainty [8]. Many studies have used test objects with known properties, commonly referred to as phantoms, to investigate the accuracy, repeatability, and reproducibility of qMRI biomarkers [9–13], and to provide a measure of uncertainty when used in conjunction with in vivo imaging [14–24]. In multi-centre clinical trials, phantoms have been used to establish a standardised imaging protocol at each site [4] and to ensure that differences in site specific protocols are within acceptable limits [25]. Additionally, phantom measurements serve a role in quality assurance (QA) throughout clinical trials, to detect changes in scanner performance [26, 27]. The development and evaluation of qMRI sequences can also be aided by phantom studies, for example to compare multiple diffusion weighted MRI (DW-MRI) sequences and select a sequence with an acceptable level of geometric distortion and a ADC accuracy [19, 20, 23, 24].

Phantoms for qMRI measurements include diffusion phantoms [28, 29] and relaxometry phantoms [30]. Diffusion phantoms are used to evaluate parameters such as ADC which is correlated to cellularity in many solid tumours [4, 31]. The isotropic diffusion phantom [28, 29] was collaboratively developed by the National Institute of Standards and Technology (NIST), National Cancer Institute (NCI), and Radiological Society of North America (RSNA). Relaxometry phantoms [30] are used to evaluate longitudinal (*T*_1_) and transverse (*T*_2_) relaxation. One application of longitudinal relaxation measurement is for the quantitative modelling of dynamic contrast enhanced (DCE) MRI, which can be used to assess changes in vasculature [32, 33]. The system phantom [30], which includes relaxometry modules and a suite of other image quality test objects, was collaboratively developed by NIST and the International Society of Magnetic Resonance in Medicine (ISMRM). Another commercially available relaxometry phantom is the Eurospin phantom [34] (Leeds Test Objects, UK).

Measurement uncertainty is a combination of uncertainty in every step of the measurement process, including the software used to generate and assess qMRI data. There are open-source [30, 35, 36] and commercial (qCal-MR™, CaliberMRI, USA) software solutions to analyse the NIST/ISMRM system phantom. Similarly, images of the NIST/NCI/RSNA diffusion phantom can be analysed with the fully automated commercial qCal-MR™ software, or a semi-automated open-source solution [37]. Semi-automated software, which requires the manual placement of regions of interest (ROI) may increase uncertainty in qMRI analysis due to a higher inter-observer variability than automated ROI detection methods [36]. Whilst commercial software offers a standardised automated solution for diffusion phantom analysis, an open-source option may enable researchers to explore novel imaging sequences and less established diffusion models [38, 39]. Additionally, there is not currently an open-source tool capable of analysing multiple techniques and phantoms.

We have updated the Magnetic Resonance BIomarker Assessment Software (MR-BIAS) [36], which was initially validated on relaxometry data, to provide a fully automated open-source solution for the analysis of both relaxation and diffusion phantom data. To validate the technical performance of the software, we compared ADC values calculated with MR-BIAS against ADC values reported independently in two previously published studies [10, 25]. The first, a longitudinal study [10] from a single scanner, was used to validate the analysis of diffusion data and to explore the impact of ROI size and position. The second, a multi-centre study [25], provided a more complex dataset to validate the diffusion analysis on images from multiple field strengths, imaging protocols, scanner manufacturers and models.

## Methods

### Image analysis software

**Fig. 1:**
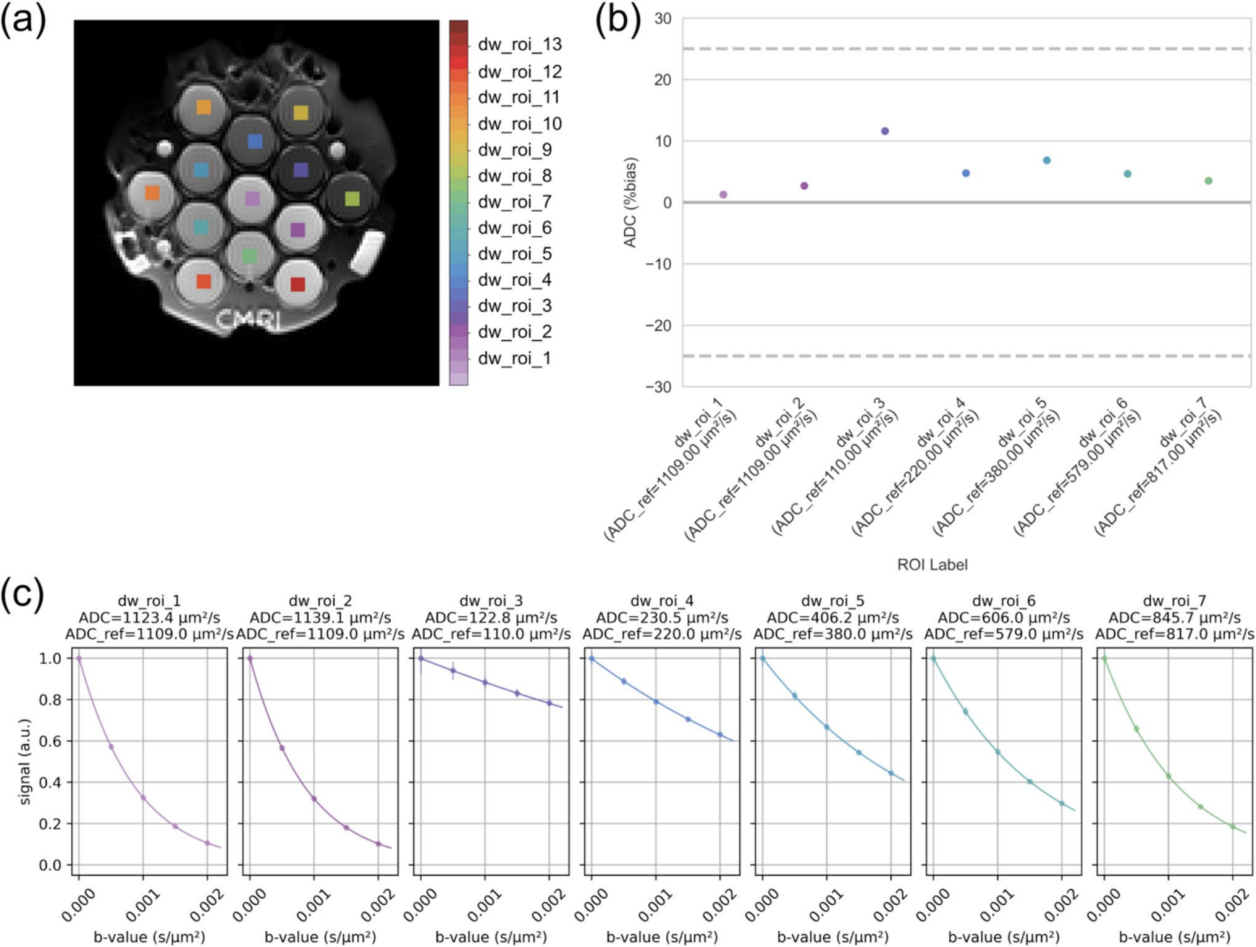
An example of the components of a PDF report generated by MR-BIAS from analysis of a NIST/NCI/RSNA diffusion phantom. The report includes **(a)** the detected ROIs on the target image, **(b)** a summary of the accuracy of estimated apparent diffusion coefficients relative to NMR reference values at the specified temperature, and **(c)** detail for each ROI of the (circular markers) measured signal and the (line) estimated exponential diffusion curve

To analyse phantom images, MR-BIAS automates four main tasks: image sorting, ROI detection, model fitting and result reporting [36]. MR-BIAS is available for download and community contribution at http://github.com/JamesCKorte/mrbias. The tool is written in Python (v3.7) and uses well established python packages for image analysis [40–44] and reporting [45, 46]. MR-BIAS was built with software extensibility principles [47] which allowed relatively straightforward modification to add support for a diffusion phantom. An example of the components of a diffusion phantom report are shown in Fig. 1, a full report is also provided for reference (see Supplementary Material). We describe the software architecture with a unified modelling language (UML) class diagram (Supplementary Figure 1). For a detailed description of the software, please refer to the original publication [36]. To reduce possible barriers to adoption, we have written several introductory tutorials, including a diffusion analysis tutorial, which are available on the open-source GitHub repository.

Updates to the software included image sorting for diffusion images from multiple manufacturers, a diffusion model, and a diffusion reference phantom. A shape-based ROI detection method was implemented (details in Supplementary Material), as the existing registration based detection could not reliably process images with the larger spatial distortions often present in diffusion weighted echo planar imaging (EPI). Manufacturer specific DICOM metadata interpretation was required to account for the variation in DICOM tag usage between manufacturers, such as differences in the encoding of image intensity values [48]. Parameter maps calculated on the MRI scanner also are now detected, allowing the reporting of metrics in detected ROIs, such as the mean and standard deviation of manufacturer calculated ADC values.

#### Diffusion modelling

Estimation of the apparent diffusion coefficient (ADC) from a series of diffusion weighted images was based on a mono-exponential model,

$$s\left( b \right) = s_{0} e^{{ - b {\text{ADC}}}}$$

which includes the intensity in a trace weighted diffusion image, $s\left( b \right)$, the intensity in an image with no diffusion weighting, $s_{0}$, and the diffusion weighting factor, $b$, commonly referred to as the *b* value. Estimation of ADC values was achieved with linear regression (SciPy [41], v1.7) of a linearised form of the model,

$$\log_{e} \left( {s\left( b \right)} \right) = - b {\text{ADC + }}\log_{e} \left( {s_{0} } \right).$$

### Evaluation of the software

The quantitative performance of the software was validated against a previously published single-centre longitudinal study [10] and a multi-centre multi-vendor study [25]. The single-centre dataset was used to validate the accuracy of ADC values generated by MR-BIAS, and to identify an optimal sized central ROI to minimise variability in ADC assessments. The multi-centre multi-vendor study provided a more complex dataset on which to validate the fully automated MR-BIAS diffusion analysis. A summary of the two datasets and key analysis parameters are provided in Table 1.

**Table 1:** A summary of the diffusion weighted datasets and analysis parameters used to validate the updated MR-BIAS software

	Single-centre longitudinal dataset	Multi-centre multi-vendor dataset
Purpose in this study	Validate accuracy of MR-BIAS ADC values Assess spatial variability of ADC and establish an optimal ROI size	Validate accuracy MR-BIAS ADC values over a range of scanner manufacturers, imaging protocols, and field strengths
Scanner		
Manufacturer	Siemens (*n* = 1)	Siemens (*n* = 5), Philips (*n* = 6), GE (*n* = 2)
Field strength	3.0 T (*n* = 1)	1.5 T (*n* = 5) and 3.0 T (*n* = 8)
Phantom		
Manufacturer	CalibreMRI	High Precision Devices
Temperature	0 °C	0 °C
Data		
Used in this study	Axial images from 12 monthly sessions, 4 repeat images per session	Axial images from 13 institutes, 4 repeat images (1 repeat only for Institute J) for both an institutional protocol and a benchmark protocol
Excluded in this study	Sagittal and Coronal images	Images from Institute F excluded as Enhanced DICOM is not currently supported by MR-BIAS As in the published study, images from Institute N institutional protocol images excluded due to temperature
Total number of images used	*n* = 48	Benchmark (*n* = 49) Institutional (*n* = 43)
Region of interest (ROI)		
Detection method	Image registration based	Shape based
Manual	Location matched exactly to published study (3 central slices, radius = 5mm)	Axial slice matched exactly to published study, axial location as detected (single slice, radius = 8mm)
Optimal	Location as detected (radius = 5mm, height = 10mm)	Location as detected (radius = 5mm, height = 10mm)
Whole Vial	Location as detected (radius = 12mm, height = 32mm)	–
Analysis method used in original publication	Custom Python script	Custom MATLAB script

Both studies acquired diffusion weighted images of NIST/NCI/RSNA diffusion phantoms, which contain thirteen vials of differing iso-tropic diffusivity, achieved with different concentrations of polyvinylpyrrolidone (PVP) including (%): 0, 10, 20, 30, 40, and 50. Measurements in both studies were made using an ice-bath to establish thermal equilibrium near 0°C, as per Quantitative Imaging Biomarker Initiative (QIBA) ADC profile [49]. Reference ADC values for the diffusion phantoms used in these studies are provided in Supplementary Table 1.

#### Single-centre longitudinal study

The single-centre dataset [10] consists of DW-MRI of a NIST/NCI/RSNA diffusion phantom (CalibreMRI, model 128, serial number 128-0113) acquired on a Siemens 3T Skyra MRI scanner during twelve monthly sessions. Diffusion weighted images were acquired with four repeats, and in three phantom orientations (axial, coronal, sagittal), during each of the monthly sessions.

The axial diffusion weighted images (*n* = 48) were analysed with MR-BIAS to calculate ADC values in a single ROI per vial. Registration based ROI detection was used as there was minimal spatial variation in the imaging across months; each set of DW-MRI was rigidly registered to a template image, a trace-weighted image from the first repeat of the first monthly session. Template ROIs, which were manually defined on the template image, were then transformed onto the DW-MRI. The diffusion model was fitted to the average signal per ROI, with trace weighted images for all b-values used in the analysis. MR-BIAS ADC values were compared to those generated from a custom python script used in the original study [10]. The original study investigated all 13 diffusion vials, however, only explicitly reported on the quantitative ADC values for the central water vial. Thus, the script was re-run to generate comparison values for all vials and each monthly repeat. Minor modifications were made to the custom script to load images from a different file system, but no changes were made to the signal pre-processing or diffusion model fitting.

Additionally, we used MR-BIAS to extract ADC values from ADC maps generated on the scanner (*n* = 48). We will refer to the scanner generated ADC values as ‘inline’ results to differentiate them from ADC values estimated by MR-BIAS or a custom script. Finally, QIBA diffusion profile metrics [49] were calculated for the central vial (0% PVP) using MR-BIAS inline ADC values and compared to the inline ADC metrics reported in the original study [10].

#### Multi-centre multi-vendor study

The multi-centre dataset [25] consists of DW-MRI of three NIST/NCI/RSNA diffusion phantoms (High Precision Devices, model 128) acquired at different field strengths (1.5 T and 3.0 T) and of different manufacturer and model (Siemens, Philips, and GE). A diffusion phantom was scanned at fourteen institutes with a benchmark protocol, using the same sequence parameters at each institute, and an institute specific protocol. The benchmark and institute specific diffusion sequences were repeated four times in a single imaging session. Images from Institute F were excluded from the analysis as MR-BIAS does not yet support enhanced DICOM data. As in the original study, Institute J had no repeat imaging and Institute N images from the institute specific protocol were excluded due to temperature deviation from 0 °C.

Diffusion weighted images from benchmark (*n* = 49) and institute specific (*n* = 43) protocols were analysed with MR-BIAS to calculate ADC values in a single ROI per vial. Shape based ROI detection was used to account for varying levels of imaging distortion, and related variability in vial location in the benchmark and institutional and imaging protocols. ADC was estimated from the average signal per ROI, using all available trace weighted images for the benchmark protocol. For the institute specific protocol analysis specific b-values (200, and 1000 s/mm^2^) were used for the majority of institutes, other than Institute J (200, 400, 800, 1000 s/mm^2^). MR-BIAS generated ADC values were compared to those from the original study [25], which were generated in a central analysis with a custom MATLAB (MathWorks, USA) script. The imaging data and the results from the central analysis are publicly available [50]. Summary ADC metrics such as the short-term repeatability coefficient (%RC) and the inter-institutional reproducibility (%RDC) were calculated on MR-BIAS ADC values in the same manner as, and then compared to, those reported in the original study [25].

#### ADC variation due to ROI size and location

To investigate the spatial variation in ADC values, a voxel-wise analysis was performed in each vial of the phantom DW-MRI from the single-site dataset only. The data output from MR-BIAS includes an estimated ADC value and a cylindrical coordinate for each voxel within an ROI. The use of cylindrical coordinates in each ROI provided a shared coordinate system for the comparison of all DW-MRI in the axial orientation (*n* = 48). The spatial variation of ADC values was then visualised with respect to radial position and height within each ROI. We then identified an optimal size ROI from these trends of ADC variation, where we define the optimal ROI as a region that encompasses a reasonable number of voxels while minimising the variation in ADC. The optimal ROI selection method is described in more detail at the end of this section.

To explore the impact of spatial variation in ADC values on phantom analysis, we assessed MR-BIAS generated ADC values and ADC summary metrics for three different sets of cylindrical ROIs; manual, optimal, and whole vial. Manually matched sets of ROIs, which we refer to as ‘manual’, were matched in size and location to those used in the previous studies to establish the equivalence of ADC estimation by MR-BIAS in the same, or similar, spatial locations. Manual ROIs for the single-site study were exactly matched to the original study (3 central slices, radius = 5mm). Manual ROIs for the multi-centre study were matched in size (single slice, radius = 8mm) and to the same axial slice as the original study, but the ROI locations in the axial plane were automatically detected by MR-BIAS. An automatically detected set of optimally sized ROIs, referred to as ‘optimal’, were used to assess ADC in a central region of low variation for each diffusion vial in both studies. A set of large ROIs (radius = 12 mm, height = 32 mm), referred to as ‘whole vial’, were used to assess ADC in regions covering the majority of each diffusion vial in the single-site study only. The whole vial and optimal ROI sets provide an assessment of ADC values generated with automatically detected ROI position.

The optimal ROI was determined by assessment of the spatial variation of ADC values from the voxel-wise analysis. To visualise the change in bias over each region of interest, we adjusted the bias to be relative to a small central region in each vial. We define relative bias as,

1 $$\begin{array}{*{20}c} {{\text{bias}}_{{{\text{relative}}}}^{i} \left( {r, h} \right) = {\text{bias}}^{i} \left( {r,h} \right) - {\text{bias}}_{{{\text{centre}}}}^{i} } \\ \end{array}$$

where $r$ is the radial distance and $h$ is the height distance from the centre of the $i$-th region of interest. The central bias, ${\text{bias}}_{{{\text{centre}}}}^{i}$, is calculated over a small central region ($r \le 1 {\text{mm, h }} \le 1 {\text{mm}}$) for each ROI, with bias defined as,

2 $$\begin{array}{*{20}c} {{\text{bias}}^{i} = \overline{Y}_{i} - X_{i} } \\ \end{array}$$

where $\overline{Y}_{i}$ is the mean estimated value over potentially repeated measurements and $X_{i}$ is the ground truth measurement for the $i$th region of interest. The size of the optimal ROI was selected as a central cylindrical region to keep the ADC variation low, specifically to achieve a $\left| {{\text{bias}}_{{{\text{relative}}}} } \right| < 5$ µm^2^/s. A single optimal ROI size was defined by visual inspection of ${\text{bias}}_{{{\text{relative}}}}^{i}$ trends, to meet the variation condition across all ROIs in the single-centre dataset.

#### Statistical analysis

To evaluate the similarity of ADC values between MR-BIAS and the published studies, ADC bias (Eq. 2) was calculated using the manufacturer provided ADC measurements at 0°C as the ground truth (see Supplementary Table 1). Equivalence of ADC bias between MR-BIAS and the published studies was evaluated with a two one-side test (TOST) [51, 52] using a paired Wilcoxon signed-rank test. A TOST was performed with SciPy (v1.7) [41] for each ROI on three different sets of images: the single-site dataset (across all months and repeats), the benchmark protocol of the multi-centre dataset (across all institutes and repeats), and the institutional protocol of the multi-centre dataset (across all institutes and repeats). For each of the sets of images, and for each of the ROI sets (manual, optimal, whole vial), the threshold for equivalence was lowered until all ROIs in an image set were significantly equivalent ($p < 0.05$).

## Results

### Single-centre longitudinal study

**Fig. 2:**
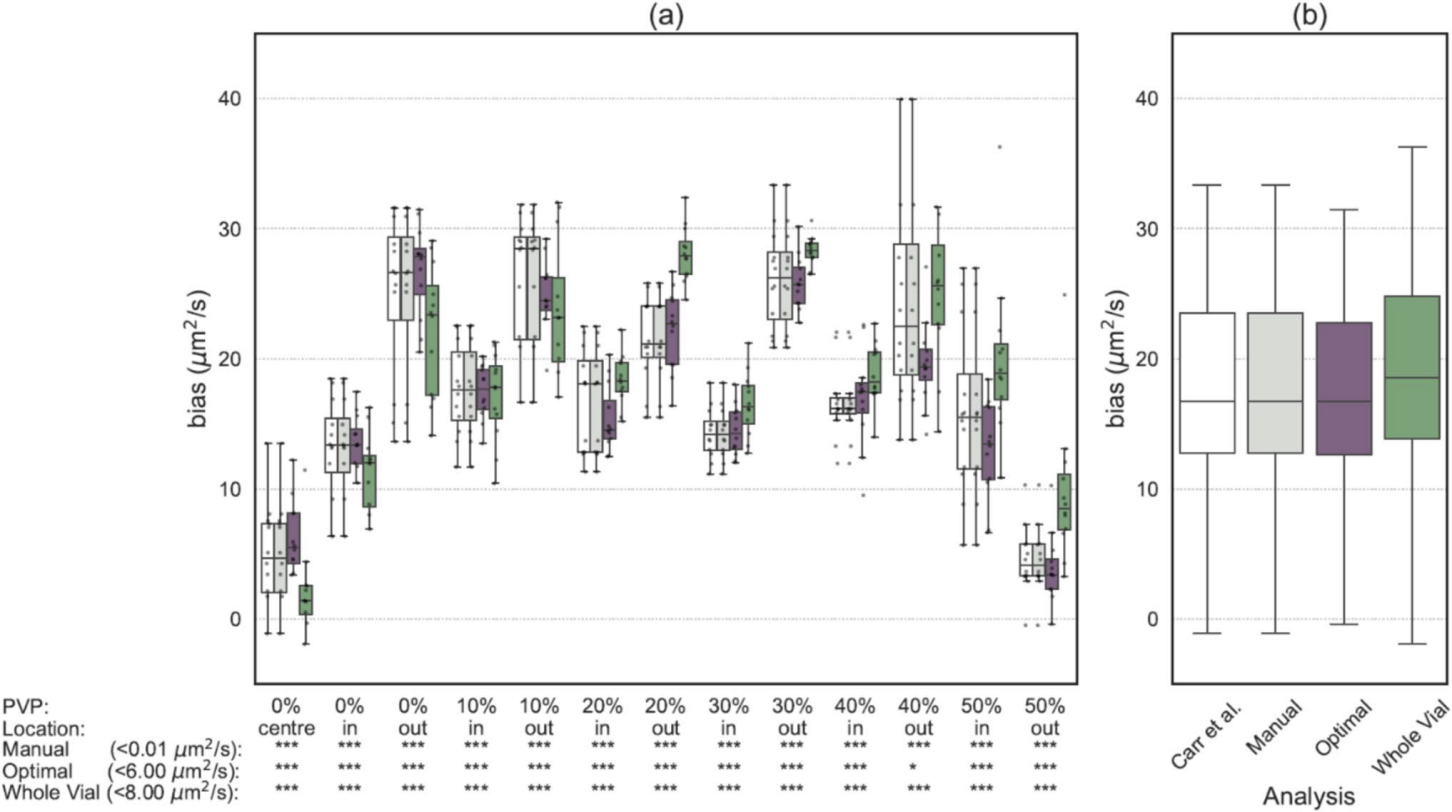
The quantitative performance of ADC values (**a**) in each vial of a diffusion phantom, and (**b**) averaged across all vials. The ADC values were calculated by (white) a custom python script from a previously published study [10] and compared to those estimated by MR-BIAS in (grey) an ROI matched in size and position to the previous study, (purple) an automatically detected ROI with an optimal size, and (green) an automatically detected ROI that covers the majority of each diffusion vial. The equivalence of MR-BIAS to the original study is listed under the x-axis (i.e. < 6 um^2^/s), where the number specifies the tolerance and the associated statistical equivalence is provided for each ROI (*p < 0.05, **p < 0.01, ***p < 0.001). The y-axis range has been set for clarity and excludes some outliers

Quantitative performance of MR-BIAS as assessed on the single-centre dataset is summarised in Fig. 2, which displays the bias (Eq. 2) as the deviation of estimated ADC values from the ADC reference values. ADC values calculated by MR-BIAS were statistically equivalent to the original study within a tolerance of ± 0.01, ± 6, and ± 8 μm^2^/s for the manually matched ROI, optimal size ROI and whole vial ROI, respectively. The QIBA metrics calculated on inline ADC values extracted by MR-BIAS (Table 2) were comparable to those from the original study, when using the manually matched ROI or an automatically detected optimal ROI. As in the original study, the QIBA metrics from manual and optimal ROIs met the QIBA conformance criteria. Many of the MR-BIAS whole vial QIBA metrics failed to meet the QIBA conformance criteria, differing from the metrics reported in the original study, which used a different spatial volume.

**Table 2:** Summary of QIBA diffusion profile metrics as reported in original study and compared to those based on inline ADC values extracted by MR-BIAS in a manual ROI, an automated ROI of optimal size, and an automated ROI covering the majority of each diffusion vial. Metrics are calculated for the central water vial from ADC maps generated on the MRI scanner

Metric	Analysis					QIBA tolerance
	Custom python script [10]		MR-BIAS			
	As published [10]	Independent calculation for this study from shared data	Manual ROI	Automated ROI (optimal)	Automated ROI (whole vial)	
$\left| {\% {\text{bias}}} \right|$	0.05	0.05	0.04	0.24	1.97	≤ 3.60
RC_ST_ (μm^2^ /ms)	0.003	0.003	0.003	0.003	0.108	≤ 0.015
CV_ST_ (%)	0.1	0.1	0.1	0.1	3.63	≤ 0.5
RC_LT_ (μm^2^/ms)	0.028	0.028	0.028	0.023	0.067	≤ 0.065
CV_LT_ (%)	0.9	0.9	0.9	0.74	2.23	≤ 2.2

**Fig. 3:**
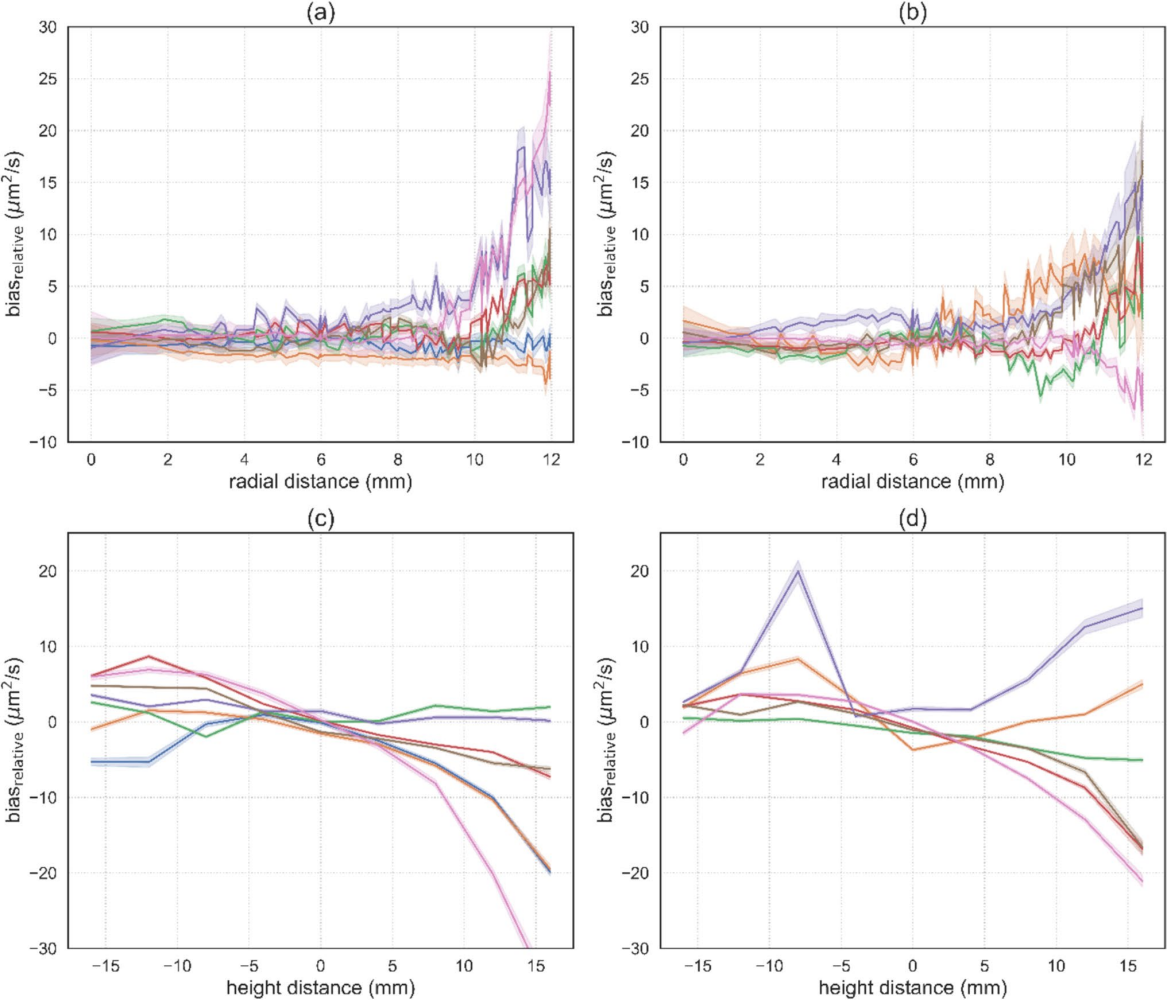
Analysis of ADC variation as a function of spatial position in a cylindrical ROI, with respect to (**a**, **b**) radial distance and (**c**, **d**) height distance from the centre of each vial. ADC values are shown in ${\text{bias}}_{{{\text{relative}}}}$, to provide the variation of bias across each vial relative to a small central region (*r* = 1.0mm, height = ± 1.0mm). PVP concentration is denoted with colour, such as (blue) 0% central vial, (orange) 0%, (green) 10%, (red) 20%, (purple) 30%, (brown) 40%, and (pink) 50%. The plots are separated into (**a**, **c**) vials in the centre or the inner ring and (**b**, **d**) vials in the outer ring. The (solid line) mean and (shaded region) 95% confidence interval are shown for all voxels at a particular radial or height distance

The analysis of spatial variation of MR-BIAS ADC in each cylindrical ROI (Fig. 3) provided trends of ADC variation relative to the central region of the ROI, as a function of radial distance and height distance from the centre of the ROI. Variation in ADC in the radial direction was low and relatively constant in the central region ($r \le 5$ mm) after which the variation increased, in some ROIs appearing to be approximately exponential with respect to radial distance. Variation in relative ADC bias in the height direction increased to one side of the centre of the ROI, and decreased in the opposing direction. For some ROIs, the ADC appears to vary approximately quadratically with respect to height. From these observations, a centrally located optimal ROI size was defined (height = 10 mm, diamete*r* = 10 mm) based on keeping the $\left| {{\text{bias}}_{{{\text{relative}}}} } \right| < 5$ μm^2^/s.

A comparison of inline ADC values extracted by MR-BIAS and those estimated by MR-BIAS in optimal ROIs are shown in Supplementary Figure 2. The inline ADC values, those extracted from ADC maps calculated on the MRI scanner, are on average 1.4 μm^2^/s lower than ADC values estimated by MR-BIAS, where the estimation is based on nominal *b* values in the DW-MRI metadata.

### Multi-centre multi-vendor study

**Fig. 4:**
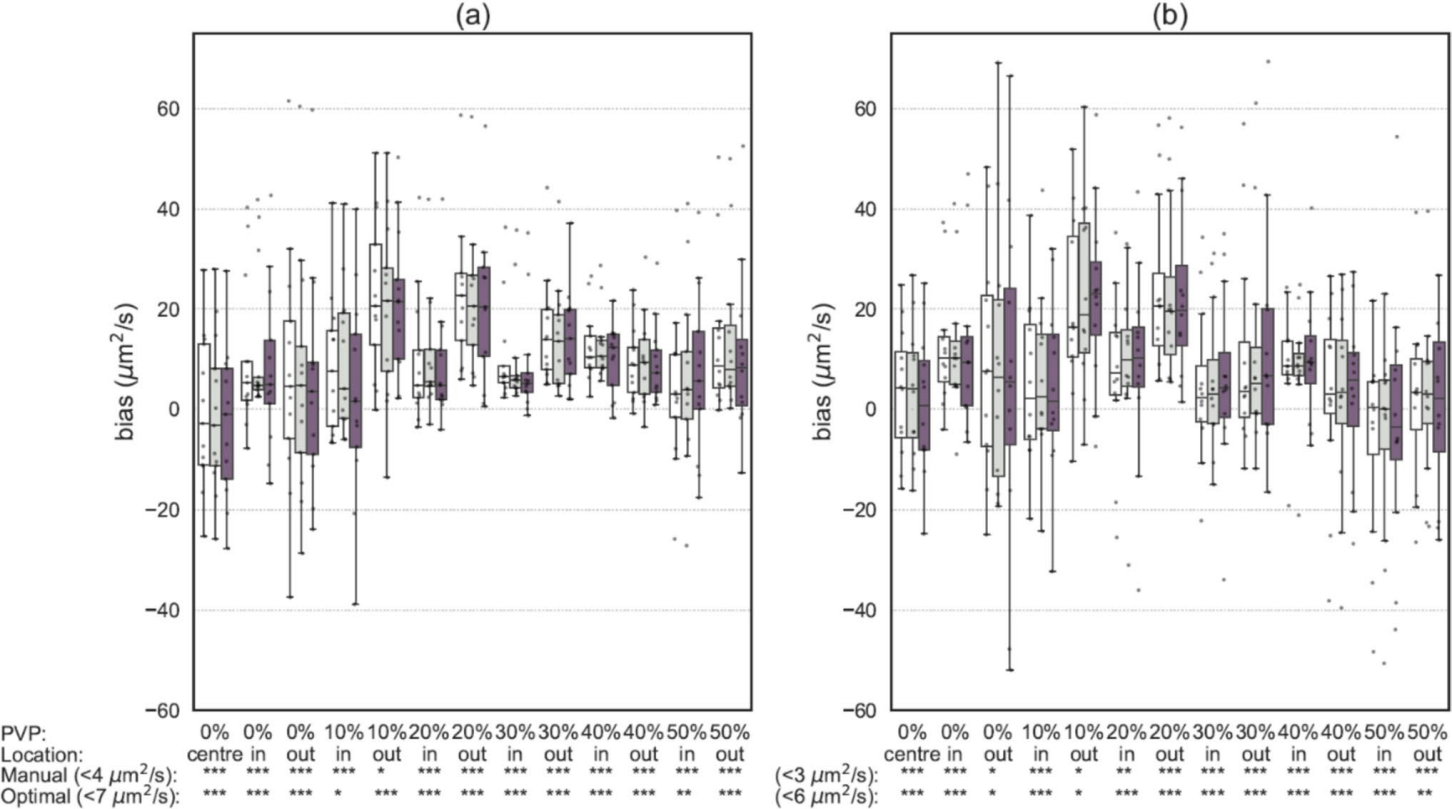
The ADC bias is provided for (**a**) the benchmark imaging protocol and (**b**) institute specific protocols. Bias in ADC values for each ROI are shown for those (white) reported in the original study [25], (grey) estimated with MR-BIAS with manually matched ROIs, and (purple) estimated with MR-BIAS with automatically detected ROIs of an optimal size. The equivalence of MR-BIAS to the original study is listed under the x-axis (i.e. < 4 um^2^/s), where the number specifies the tolerance and the associated statistical equivalence is provided for each ROI (*p < 0.05, **p < 0.01, ***p < 0.001). The y-axis range has been set for clarity and excludes some outliers

Quantitative performance of MR-BIAS as assessed on the multi-centre multi-vendor dataset is summarised in Fig. 4. ADC values calculated by MR-BIAS with a manually matched ROIs were statistically equivalent to ADC values previously reported within a tolerance of ± 4, and ± 3 μm^2^/s for the benchmark and institutional protocols, respectively. Automated MR-BIAS analysis with optimal ROIs generated ADC values which were statistically equivalent to those previously reported within a tolerance of ± 7, and ± 6 μm^2^/s for the benchmark and institutional protocols, respectively.

Summary ADC metrics reported in the original study are compared to those based on ADC values generated by MR-BIAS in Table 3. The range of bias across institutes on the benchmark protocol were 1–38 μm^2^/s for manually matched ROIs, and − 5 to 37 μm^2^/s for automatically detected optimal ROIs. The range of bias as calculated on MR-BIAS ADC values was comparable to the published study, which reported a bias range of 2 to 40 μm^2^/s. Similarly, for images from the institute specific protocol, the range of bias in MR-BIAS ADC values was − 8 to 29 μm^2^/s and − 8 to 37 μm^2^/s for manual ROIs and optimal ROIs, respectively. The bias calculated on MR-BIAS ADC values was comparable for the manual ROIs, and slightly higher for the optimal ROIs, when compared to the bias range of − 7 to 29 μm^2^/s reported in the original study.

**Table 3:** Summary metrics as reported in the previously published study, compared to those calculated from MR-BIAS estimated ADC values from manually matched ROIs and automatically detected ROIs of an optimal size

Dataset	Metric		ADC Analysis			
			Centralised MATLAB script [25]		MR-BIAS	
			As published [25]	Independent calculation for this study on published ADC data [50]	Manual ROI	Automated ROI (Optimal)
Bench.	${\text{bias }}$ (μm^2^/s)	Min	2	1	1	− 5
		Max	40	39	38	37
Inst.	${\text{bias }}$ (μm^2^/s)	Min	− 7	− 8	− 8	− 8
		Max	29	28	29	37
	%RC (%)	Median	3	3	3	3
		Range	1–13	1–13	1–7	1–10
	%RDC (%)		18	18	18	27

The short-term repeatability coefficient (%RC) as calculated on MR-BIAS ADC from the institute specific protocols had a (median, minimum–maximum) of 3%, 1–7% for manually matched ROIs and 3%, 1–10% for automatically detected optimal ROIs. The %RC calculated from MR-BIAS was comparable to the 3%, 1–13% reported in the original study. The inter-institutional reproducibility (%RDC) as calculated on MR-BIAS ADC from the first repeat of the institute specific protocols was 18% for manual ROIs and 27% for optimal ROIs, in comparison to the 18% reported in the original study.

## Discussion

In this study, we have demonstrated that MR-BIAS can automatically analyse diffusion weighted phantom images and estimate robust ADC values in all regions of interest. In addition to statistical equivalence of ADC values, this study demonstrates that MR-BIAS can reach the same study outcomes as the custom scripts used in the original studies [10, 25]. When using ROIs with a manually matched spatial location, the tolerance for statistical equivalence of ADC values between MR-BIAS and the published studies was low for both the single-centre study (± 0.01 μm^2^/s) and the multi-centre study for the benchmark protocol (± 4 μm^2^/s) and institutional protocol (± 3 μm^2^/s). These results suggest that MR-BIAS could be a useful tool for phantom-based quality assurance in a multi-centre setting. The slightly higher tolerance for equivalence in the multi-centre study is attributed to variation in ROI locations in the axial plane, as MR-BIAS automatically detected the in-plane location on an axial slice which was manually matched to the original study.

This study showed that when using automatically detected ROIs, a higher tolerance for equivalence was required for ROIs of optimal size for the single-site study (± 6 μm^2^/s) and the multi-centre study for the benchmark protocol (± 7 μm^2^/s) and institutional protocol (± 6 μm^2^/s). The highest tolerance for equivalence was observed when using automatically detected ROIs which covered the whole vial for the single-centre study (± 8 μm^2^/s). A larger tolerance for equivalence when using an automated ROI is due to differences in the voxels included in each ROI, as compared to the original studies. In the case of a whole vial ROI, the assessed region includes areas at the periphery of the vial that have larger variations in ADC, as shown in Fig. 3. These findings show us that ROI placement does influence the assessment of ADC on diffusion phantom images. Our previous study of relaxometry phantoms suggests that automated ROI placement is preferable to manual ROI placement, as it is more efficient and reduces the inter-observer variability [36].

Our analysis of spatial variation of ADC provides insight into the variation of ADC values in the radial and height directions of cylindrical ROIs. Larger variation was observed in the height direction, which is also the longitudinal direction, which is along the bore of the MRI scanner. Our observations are similar to a previous study using a homogeneous diffusion phantom which reported quadratic variations in the longitudinal direction, with errors in ADC of up to 55% in the longitudinal direction, and 25% across the scanner bore [53]. Another study reported 24% bias in ADC when the phantom was offset by 8 cm from the isocentre of a 1.5T scanner [15]. Variations in ADC are attributed to gradient non-linearity (GNL) leading to spatial variations in b-value [15, 53], for which there are a number of proposed correction methods [54–56]. One study reported on a vendor correction available on a MRI scanner [17], which suggests that correction for GNL in inline ADC maps may be more commonly available in the future. This work demonstrates that MR-BIAS can analyse the spatial variation of ADC values, a feature which could be useful for the evaluation of GNL correction methods.

We also observed differences between inline ADC values as calculated on the scanner and MR-BIAS ADC values estimated from DW-MRI (Supplementary Figure 2). This was also noted in the original single-centre study [10] and in a previous study [57], which attributed the differences in ADC to more accurate b-values being used on the scanner, as opposed to the nominal b-values reported in the DICOM metadata which are often used for ADC estimation. Given these differences in ADC values estimated from DW-MRI and manufacturer calculated ADC maps, and the potential additional differences in ADC maps from vendor GNL corrections in the future, we recommend that phantom QA software should support the analysis of manufacturer derived ADC maps, as well as the estimation of ADC from a diffusion model.

Conformance with the QIBA diffusion profile was demonstrated with MR-BIAS ADC values generated from the single-centre dataset [10], when using ROIs with a manually matched location or with automatically detected ROIs with an optimal size. The automated analysis of the multi-centre multi-vendor dataset [25] demonstrates that MR-BIAS can reliably generate summary metrics for a wide range of MRI scanners and imaging protocols. If the single-centre study had been analysed using whole vial ROIs, the scanner would have been reported as not compliant with the QIBA profile. This highlights the need for a judicious choice of ROI, selecting a region that is large enough to include a reasonable number of voxels for a robust estimation of ADC, whilst avoiding peripheral regions of the diffusion vials that have an increased variability in ADC. Whilst we defined an optimal ROI to be one which keeps ADC variation to a minimum, it could be argued that a whole vial ROI may report a wider range of ADC variation, which may be more representative of the larger variation observed in in vivo DW-MRI studies [19, 20].

Our selection of a ${\text{bias}}_{{{\text{relative}}}}$ threshold of 5 µm^2^/s was based on the observed magnitude of ${\text{bias}}_{{{\text{relative}}}}$ in the voxelwise analysis (Fig. 3), and to be relatively small in comparison to the 100th percentile of ADC bias over repeated single scanner measurements (~ 40 µm^2^/s) [10], and across different scanner field strengths and manufacturers (~ 50 µm^2^/s) [25]. Our definition of an optimal ROI size (radius and height) from visual inspection of the spatial variation trends (Fig. 3) is based on a single scanner dataset, but when a more variable multi-vendor dataset was analysed with the same optimal ROI size the results were comparable to those of a manually placed ROI in the original study [25]. This suggests that shape-based detection with the proposed optimal ROI size is relatively robust for datasets with moderate spatial distortion and minimal intensity variation near the centre of each vial. Alternatively, for users wanting to customise the ROI size, we recommend they perform a similar voxelwise analysis to inform their selection of an ROI size which is suitable for their data.

A number of features were added to MR-BIAS during this study, such as shape-based ROI detection for the NIST/NCI/RSNA diffusion phantom, which removes the need for creating a new registration template for different imaging protocols. An alternative method to address the spatial uncertainty in diffusion weighted EPI images could be to apply distortion correction prior to analysis with MR-BIAS, using existing tools such as the FMRIB Software Library (FSL) eddy algorithm [58]. Shape-based detection could also be implemented in MR-BIAS for the NIST/ISMRM system phantom, like that described for ROI detection by a real-time QA software [35]. Additionally, the software was updated to support DICOM from multiple vendors, but currently only supports the inter-operability DICOM format. The ability to analyse imaging data in the enhanced DICOM format should be considered as an option for MR-BIAS in the future. This study was performed with two phantom datasets acquired at 0°C, but room temperature measurements are expected to become more common due to newer diffusion phantoms including MR visible thermometers [59, 60]. Support for MR thermometer detection and correction for ADC temperature dependence [61] should be considered in future updates of the software.

We have previously validated MR-BIAS for analysis of the NIST/ISMRM system phantom [36], with this study validating the software for analysis of the NIST/NCI/RSNA diffusion phantom. The software has also been modified to analyse the Eurospin phantom [34] for relaxometry, which could allow a similar validation to the one presented here to be performed with the Eurospin phantom relaxometry data that is publicly available from the same multi-centre multi-vendor study [25, 50]. Finally, further analysis of the public dataset with respect to b-value selection and spatial inhomogeneities could provide useful information on the variability of ADC values between manufacturers and field strengths.

## Conclusions

MR-BIAS as an open-source tool for the fully automated analysis of diffusion and relaxometry phantom data, as validated by this study and a prior study [36], respectively. This study has demonstrated the suitability of the software for use in multi-centre and multi-vendor clinical trials involving quantitative MRI biomarkers. MR-BIAS is freely available for download and community contribution at http://github.com/JamesCKorte/mrbias.

## Supplementary Information

Below is the link to the electronic supplementary material.

Supplementary file1 (DOCX 15 kb)

Supplementary file2 (PDF 149 kb)

Supplementary file3 (PDF 33 kb)

Supplementary file4 (DOCX 21 kb)

Supplementary file5 (PDF 15592 kb)

Supplementary file6 (CSV 85 kb)

Supplementary file7 (CSV 44 kb)

Supplementary file8 (CSV 313034 kb)

Supplementary file9 (CSV 286 kb)

Supplementary file10 (CSV 180 kb)

Supplementary file11 (CSV 180 kb)

Supplementary file12 (PY 13 kb)

Supplementary file13 (PY 5 kb)

Supplementary file14 (PY 20 kb)

Supplementary file15 (PY 4 kb)

## Data Availability

The authors declare that the data supporting the findings of this study are available within the paper and its supplementary information files. The single-centre dataset is available upon reasonable request to the author of the original publication (DOI: 10.1002/mp.15645). The multi-centre dataset is publicly available (10.5281/zenodo.14135025).
