# Supplementary material for: Open-source quality assurance for multi-parametric MRI: a diffusion analysis update for the magnetic resonance biomarker assessment software (MR-BIAS)
Source: MAGMA. 2025 Apr 26;38(4):639–51. doi: 10.1007/s10334-025-01252-4 (PMC12443916; doi:10.1007/s10334-025-01252-4)
Supplement: Supplementary file 1 — Supplementary file1 (DOCX 15 kb) [file 10334_2025_1252_MOESM1_ESM.docx]

| **PVP Concentration (%)** | **Location In Phantom** | **High precision devices, Diffusion phantom  (model 128)** | | **CalibreMRI,  Diffusion phantom  (model 128)** | |
| --- | --- | --- | --- | --- | --- |
|  |  | **Reference ADC (um^2^/s)** | **Uncertainty (um^2^/s)** | **Reference ADC (um^2^/s)** | **Uncertainty (um^2^/s)** |
| 0 | Centre | 1109.3 | 12.9 | 1109 | 8.3 |
|  | Inner Ring | 1104.5 | 14.9 |  |  |
|  | Outer Ring | 1117.5 | 14.9 |  |  |
| 10 | Inner Ring | 839.9 | 10.2 | 817 | 6.3 |
|  | Outer Ring | 832.9 | 10.4 |  |  |
| 20 | Inner Ring | 600.8 | 7.5 | 579 | 5 |
|  | Outer Ring | 599.3 | 7.6 |  |  |
| 30 | Inner Ring | 401.7 | 5.9 | 380 | 3.7 |
|  | Outer Ring | 402.0 | 5.3 |  |  |
| 40 | Inner Ring | 238.9 | 6.2 | 220 | 2.3 |
|  | Outer Ring | 244.2 | 5.5 |  |  |
| 50 | Inner Ring | 129.3 | 9.3 | 110 | 1.7 |
|  | Outer Ring | 124.7 | 6.9 |  |  |

Supplementary Table 1: Reference ADC values provided by phantom manufacturers for different PVP concentrations at 0°C. High precision devices values are taken from the Diffusion Phantom Model 128 Instructions (Version 2, 2016). CalibreMRI phantoms are manufactured with NIST traceable materials, ADC values are available online on request at <https://qmri.com/>. Uncertainty values represent one standard deviation of variation in the measurements.
