# Supplementary material for: Open-source quality assurance for multi-parametric MRI: a diffusion analysis update for the magnetic resonance biomarker assessment software (MR-BIAS)
Source: MAGMA. 2025 Apr 26;38(4):639–51. doi: 10.1007/s10334-025-01252-4 (PMC12443916; doi:10.1007/s10334-025-01252-4)

Supplementary Figure 1: A UML Class Diagram to outline the object-oriented structure of the MR-BIAS software. The extensibility of the software is achieved using (dashed line box) polymorphic/abstract classes. For example, the "ScanSessionAbstract" class is an abstract class which defines the general required functionality of a ScanSession object, the specific functionality is then implemented in (solid line box) concrete classes. This allows for images from different MRI scanners to be sorted using different strategies via different classes such as "SystemSessionSiemensSkyra" or "DiffusionSessionGEOptima". This design pattern creates extensibility, allowing the addition of new scanners in the future by adding a (green line box) future class such as "SystemSession...", with minimal modification to other aspects of the program. The pattern is used throughout the software to promote modification to include new MRI scanners, curve fitting models, detection methods, regions of interest and phantoms.

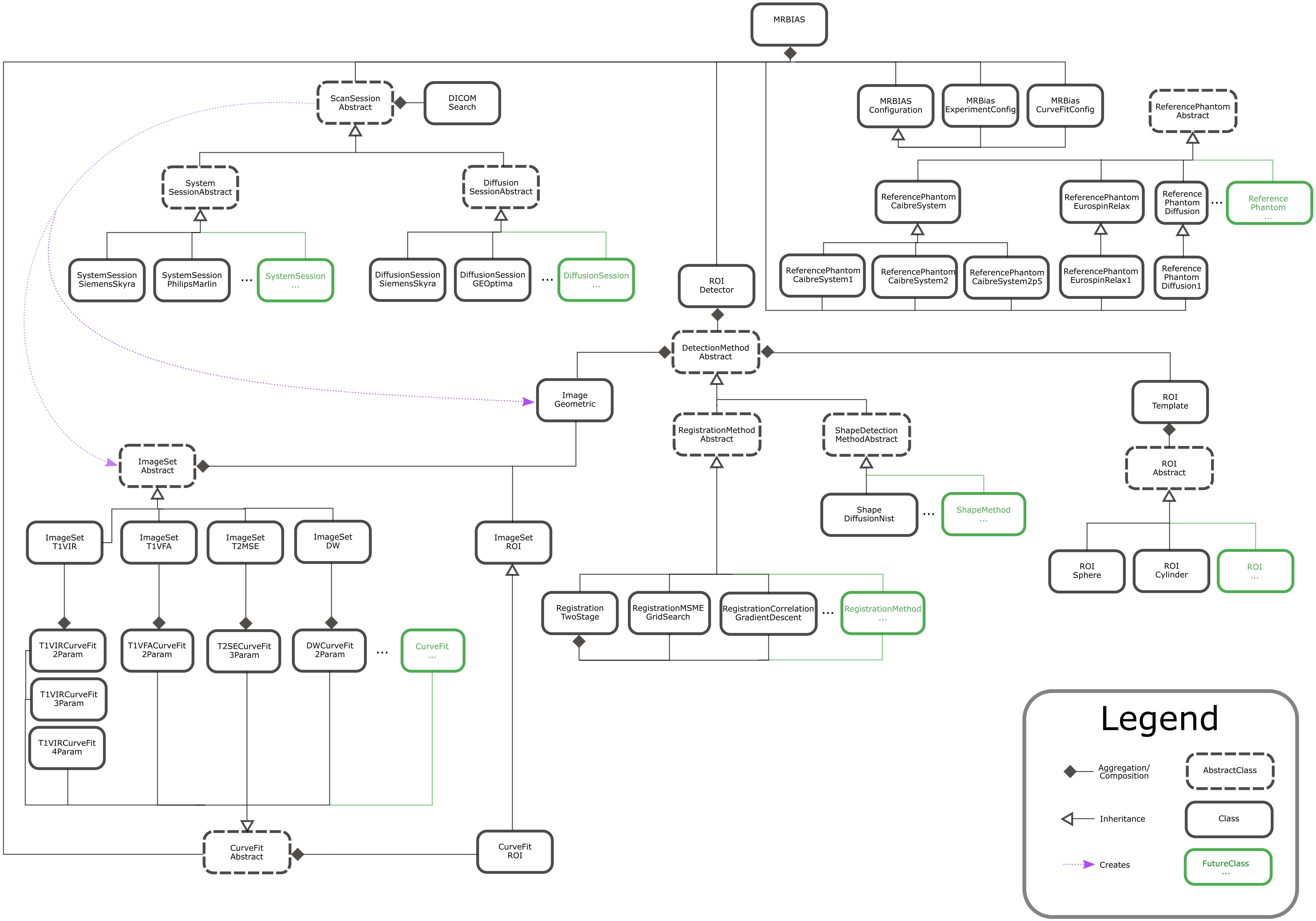

Supplement: Supplementary file 2 — Supplementary file2 (PDF 149 kb) [file 10334_2025_1252_MOESM2_ESM.pdf]
