# Supplementary figures and images for: Open-source quality assurance for multi-parametric MRI: a diffusion analysis update for the magnetic resonance biomarker assessment software (MR-BIAS)
Source: MAGMA. 2025 Apr 26;38(4):639–51. doi: 10.1007/s10334-025-01252-4 (PMC12443916; doi:10.1007/s10334-025-01252-4)

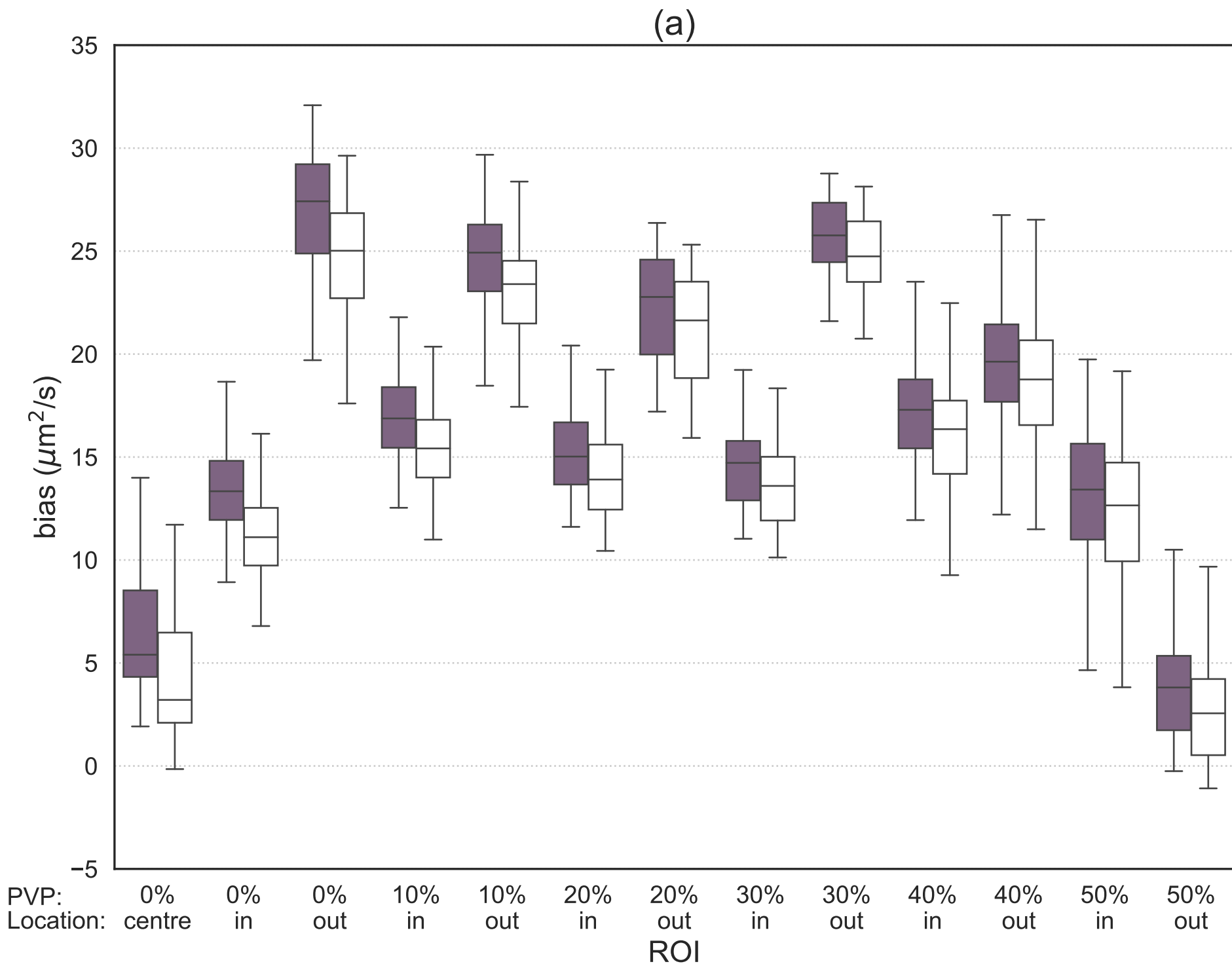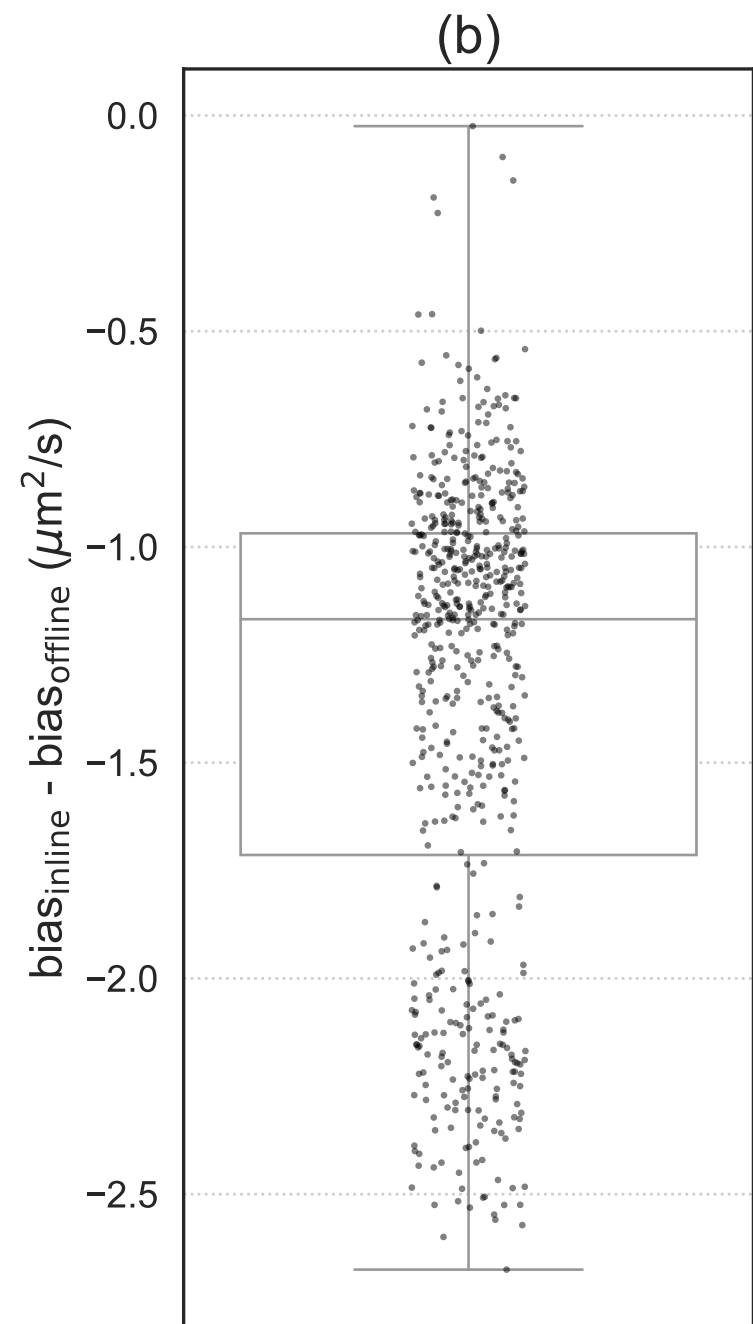

Supplement: Supplementary file 3 — Supplementary file3 (PDF 33 kb) [file 10334_2025_1252_MOESM3_ESM.pdf]
