# Supplementary material for: Open-source quality assurance for multi-parametric MRI: a diffusion analysis update for the magnetic resonance biomarker assessment software (MR-BIAS)
Source: MAGMA. 2025 Apr 26;38(4):639–51. doi: 10.1007/s10334-025-01252-4 (PMC12443916; doi:10.1007/s10334-025-01252-4)
Supplement: Supplementary file 4 — Supplementary file4 (DOCX 21 kb) [file 10334_2025_1252_MOESM4_ESM.docx]

# Diffusion Shape Based ROI detection

This document provided as conceptual guide to the shape-based detection algorithm used by MR-BIAS to detect an ROI in each of the thirteen diffusion vials of the NIST/NCI/RSNA diffusion phantom. The implementation of the algorithm is available at <https://github.com/JamesCKorte/mrbias/blob/main/mrbias/roi_detection_methods/shape_diffusion_nist.py>.

- **Assumptions**
  1. The phantom is aligned with the image grid (the bottles are aligned vertically)
  2. The axial slices of the phantom are in the first image dimension
- **Inputs to algorithm**
  1. Target image: which includes PCV bottles to detect
  2. Template image: with relative image intensities in the PVC bottles which are similar to the relative image intensities in the target image
  3. Template ROIs: which define regions on the template image, the positions of these regions will be translated onto the target image, maintaining the same offset relative to detected bottle centroids

**Detection Algorithm**

1. Detect diffusion bottles and centre of array of bottles (on both template and target image)
   1. Loop over the slices and in each slice look for circles
      1. Canny edge detector to prepare images for circle detection
      2. Hough transform to detect circles within a radius range
         1. Find circles expected for the bottles
         2. Find circles expected for the bottle caps
      3. Store the circle detection data for future steps
         1. Build up a 3D accumulation map from the bottle size circle detection
         2. Store an array of detected centre coordinates, and accumulation value
   2. Use the detected circles to identify candidate bottle locations (in-plane)
      1. Make use of the expectation bottle length, that we see circle detections in columns/lines where a bottle is
      2. Gaussian blur the 3D map of potential circle centres
         1. The blur kernel is of similar shape to the expected bottle, longer in one axis than the other
      3. Flatten/collapse the 3D map in the bottle axis direction via summation
      4. Detect candidate peaks from the resulting 2D map
         1. Local max peak detection using scikit-image, which allows for
            1. Minimum distance between peaks, which is set to the minimum expected bottle radius

Radius is used (rather than diameter) as the peak detection distance is a 1-norm not a 2-norm

- - - - 1. Threshold of absolute values to reject noise, this is set as a percent of the maximum peak accumulation (i.e. 40-50%)
  1. Estimate the bottle array location in the through plane direction using the candidate bottle locations
     1. Filter the detected circle centroids from step 1a to only include those which are close to a candidate bottle location
        1. This is done with a 2D Euclidean distance (in-plane) and a closeness threshold (<= ~4 mm)
     2. Flatten/collapse the filtered centroids in the in-plane direction to get a 1D projection of bottle locations in the through plane direction
        1. This profiles provide a distribution of the
           1. The bottle(s) extent as a function of slice number
           2. The cap(s) extent as a function of slice number
     3. Estimate the bottle and cap positions in the through slice direction
        1. Detect bottle position by fitting a rectangular function to the bottle profile, assuming an expected bottle length
        2. Detect bottle-cap position by fitting a rectangular function to the bottle-cap profile, assuming an expected cap length
        3. Jointly detect the bottle and cap position by fitting two rectangular functions to the profiles, assuming expected bottle and cap lengths & that the bottle and cap are connected
           1. Identify which end the cap is on the bottle from the single profile fits, and use this knowledge in the joint cost function
  2. Use the detected bottle location to estimate the phantom location
     1. Use the detected bottle extent to select central image slices of the phantom
     2. Average in the slice direction to create a 2D intensity map of the phantom
     3. Estimate the location of the phantom
        1. Use an Otsu method to automatically detect a threshold for foreground and background voxels
        2. Create a binary map using the Otsu threshold and detect regions using scikit-image.measure region_props
        3. Calculate the phantom centroid as the centre of mass of the largest detected region (not the weighted centre of mass)
  3. Use the detected bottle location and estimated phantom location to refine candidate bottle locations (in-plane). This allows the rejection of detected circles outside of the phantom and above/bellow the bottles.
     1. Use the detected bottle extent to crop the slices of the 3D accumulation map from step 1a
     2. Sum the cropped extent 3D accumulation map in the slice direction to create a 2D accumulation map
     3. Crop the 2D accumulation map to the detected phantom region (a circular region at the detected centre of mass)
     4. Gaussian blur the 2D map of potential circle centres
     5. Local max peak detection using scikit-image, which allows for
        1. Minimum distance between peaks, which is set to the minimum expected bottle radius
           1. Radius is used (rather than diameter) as the peak detection distance is a 1-norm not a 2-norm
     6. Select the best 13 detected peaks (as there are 13 bottles in the phantom)
  4. Detect the centre of the array of bottles based on the refined candidate bottle positions and estimated phantom location
     1. Generate a 2D bottle centre accumulation map from the candidate centres from step 1e.
     2. Use Hough transform on the centre accumulation map to look for the inner and outer ring of bottles
        1. Combine the potential centres from the inner and outer rings into a 2D centre of array accumulation map
     3. Gaussian blur the centre of array map
     4. Local max peak detection using scikit-image
        1. With a threshold of 50% the max peak to reject weaker peaks
     5. Calculate the Euclidean distance between the estimated phantom centre and each of detected peaks
     6. Use a combined cost function of distance and accumulation strength to select the best performing peak as the centre of the array of bottles
     7. Check the candidate centres from step 1e. and if any are within a threshold distance (10 mm) to the best performing peak, then used the candidate centre as the central point of the array of bottles, as it is based on a bottle detection (rather than an array detection which may have distortion effects)
  5. Use the detected centre of bottle array to identify candidate bottles in the inner ring
     1. Use a threshold distance from the expected ring diameter and a band around this (+-5mm) to select the inner ring bottle centres

1. Align the detected target bottle array with the detected template bottle array
   1. Get both images into a shared coordinate space for comparison
      1. Centre the template/fixed image on detected centre of array of bottles
      2. Centre the target/moving image on detected centre of array of bottles
         1. If required, flip the moving image to get it’s bottlecaps pointing in the same direction as the fixed image
            1. Also transform the detected bottle centres to the new coordinate system
   2. Find the optimal rotation of the moving data based on image intensity similarity and detected centroid proximity (seems more reliable using the inner ring bottles only)
      1. Extract the intensities in circular regions of the fixed image
      2. For a range of angles over a full rotation
         1. Rotate the image, extract intensities in the same circular regions, compare image similarity with a 2-norm
         2. Rotate the moving centroids and calculate a distance between nearest fixed centroid (using a kd-tree) then take the 2-norm of the vector of distances for each centroid
      3. Normalise the distance and image intensity metric vectors (over all rotations) and combine into to a joint cost functions.
         1. Define the optimal angle from the minimum of the joint cost function
   3. Rotate the moving image by the optimal angle, to align it with the fixed image
2. Associate template ROI labels with the detected bottles on the target image
   1. Associate template ROI labels with the detected bottles on the template/fixed image
   2. Match the ROI labels in the template to the detected bottles in the moving image
      1. match centroids between moving and fixed images based on distance
      2. if the distance is too far away (>20mm) then use the template expected location instead
