# Supplementary material for: Open-source quality assurance for multi-parametric MRI: a diffusion analysis update for the magnetic resonance biomarker assessment software (MR-BIAS)
Source: MAGMA. 2025 Apr 26;38(4):639–51. doi: 10.1007/s10334-025-01252-4 (PMC12443916; doi:10.1007/s10334-025-01252-4)
Supplement: Supplementary file 5 — Supplementary file5 (PDF 15592 kb) [file 10334_2025_1252_MOESM5_ESM.pdf]

```
==================================================================================================================================
     ___           ___                                             ___           ___
    /__/\         /  /\                 _____        ___          /  /\         /  /\
   |  |::\       /  /::\               /  /::\      /  /\        /  /::\       /  /:/_
   |  |:|:\     /  /:/\:\             /  /:/\:\    /  /:/       /  /:/\:\     /  /:/ /\
 __|__|:|\:\   /  /:/~/:/            /  /:/~/::\  /__/::\      /  /:/~/::\   /  /:/ /::\
/__/::::| \:\ /__/:/ /:/___  ____  /__/:/ /:/\:| \__\/\:\__  /__/:/ /:/\:\ /__/:/ /:/\:\
\  \:\~~\__\/ \  \:\/:::::/ /____/\ \  \:\/:/~/:/    \  \:\/\ \  \:\/:/__\/ \  \:\/:/~/:/
 \  \:\        \  \::/~~~~  \____\/  \  \::/ /:/      \__\::/  \  \::/       \  \::/ /:/
  \  \:\        \  \:\                \  \:\/:/       /__/:/    \  \:\        \__\/ /:/
   \  \:\        \  \:\                \  \::/        \__\/      \  \:\         /__/:/
    \__\/         \__\/                 \__\/                     \__\/         \__\/

                                        MR-BIAS v1.1.0 (released on 6th December 2024)
                                    Source code: http://github.com/JamesCKorte/mrbias
==================================================================================================================================
```

```
------------------------------------------------------------------------------------------
Please cite the following publication (for relaxometry):
------------------------------------------------------------------------------------------
      TITLE: "Magnetic resonance biomarker assessment software (MR-BIAS): an
               automated open-source tool for the ISMRM/NIST system phantom"
    AUTHORS: James C Korte, Zachary Chin, Madeline Carr, Lois Holloway, Rick Franich
    JOURNAL: Physics in Medicine & Biology
       YEAR: 2023
        DOI: https://doi.org/10.1088/1361-6560/acbcbb
------------------------------------------------------------------------------------------


------------------------------------------------------------------------------------------
Please cite the following publication (for diffusion):
------------------------------------------------------------------------------------------
      TITLE: "Open-source quality assurance for multi-parametric MRI: a diffusion analysis
               update for the magnetic resonance biomarker assessment software (MR-BIAS)"
    AUTHORS: James C Korte, Stanley A Norris, Madeline E Carr, Lois Holloway, Glenn D Cahoon
             Ben Neijndorff, Petra van Houdt, Rick Franich
    JOURNAL: UNDER REVIEW
       YEAR: UNDER REVIEW
        DOI: https://doi.org/TBD
------------------------------------------------------------------------------------------
```

Image Sorting : Summary

SERIES NUM.	DATE	TIME	DESCRIPTION	CATEGORY	IMAGE SET	GEOM SET	REF GEOM.	REF IMSET.	SERIES_UID
3001	20241010	115803	ep2d_diff_1_TRACEW	dw	dw_000	g_000	g_000		1.2.840.113704....401565800000393
3002	20241010	115803	ep2d_diff_1_TRACEW	dw	dw_000		g_000		1.2.840.113704....401565800000446
3003	20241010	115803	ep2d_diff_1_TRACEW	dw	dw_000		g_000		1.2.840.113704....401565800000499
3004	20241010	115803	ep2d_diff_1_TRACEW	dw	dw_000		g_000		1.2.840.113704....401565800000552
3005	20241010	115803	ep2d_diff_1_TRACEW	dw	dw_000		g_000		1.2.840.113704....401565800000605
4001	20241010	115803	ep2d_diff_1_ADC	adc	adc_000		g_000	dw_000	1.2.840.113704....401565800000658
5001	20241010	120157	ep2d_diff_2_TRACEW	dw	dw_001		g_000		1.2.840.113704....401565800000711
5002	20241010	120157	ep2d_diff_2_TRACEW	dw	dw_001		g_000		1.2.840.113704....401565800000764
5003	20241010	120157	ep2d_diff_2_TRACEW	dw	dw_001		g_000		1.2.840.113704....401565800000817
5004	20241010	120157	ep2d_diff_2_TRACEW	dw	dw_001		g_000		1.2.840.113704....401565800000870
5005	20241010	120157	ep2d_diff_2_TRACEW	dw	dw_001		g_000		1.2.840.113704....401565800000923
6001	20241010	120157	ep2d_diff_2_ADC	adc	adc_001		g_000	dw_001	1.2.840.113704....401565800000976
7001	20241010	120550	ep2d_diff_3_TRACEW	dw	dw_002		g_000		1.2.840.113704....401565800001029
7002	20241010	120550	ep2d_diff_3_TRACEW	dw	dw_002		g_000		1.2.840.113704....401565800001082
7003	20241010	120550	ep2d_diff_3_TRACEW	dw	dw_002		g_000		1.2.840.113704....401565800001135
7004	20241010	120550	ep2d_diff_3_TRACEW	dw	dw_002		g_000		1.2.840.113704....401565800001188
7005	20241010	120550	ep2d_diff_3_TRACEW	dw	dw_002		g_000		1.2.840.113704....401565800001241
8001	20241010	120550	ep2d_diff_3_ADC	adc	adc_002		g_000	dw_002	1.2.840.113704....401565800001294
9001	20241010	120944	ep2d_diff_4_TRACEW	dw	dw_003		g_000		1.2.840.113704....401565800001347
9002	20241010	120944	ep2d_diff_4_TRACEW	dw	dw_003		g_000		1.2.840.113704....401565800001400
9003	20241010	120944	ep2d_diff_4_TRACEW	dw	dw_003		g_000		1.2.840.113704....401565800001453
9004	20241010	120944	ep2d_diff_4_TRACEW	dw	dw_003		g_000		1.2.840.113704....401565800001506
9005	20241010	120944	ep2d_diff_4_TRACEW	dw	dw_003		g_000		1.2.840.113704....401565800001559
10001	20241010	120944	ep2d_diff_4_ADC	adc	adc_003		g_000	dw_003	1.2.840.113704....401565800001612
11001	20241010	121302	resolve_3scan...160_p2_TRACEW	dw	dw_004	g_001	g_001		1.2.840.113704....401565800001665
11002	20241010	121302	resolve_3scan...160_p2_TRACEW	dw	dw_004		g_001		1.2.840.113704....401565800001740
12001	20241010	121302	resolve_3scan...ra_160_p2_ADC	adc	adc_004		g_001	dw_004	1.2.840.113704....401565800001815

ROI Detection: Summary <g_000>

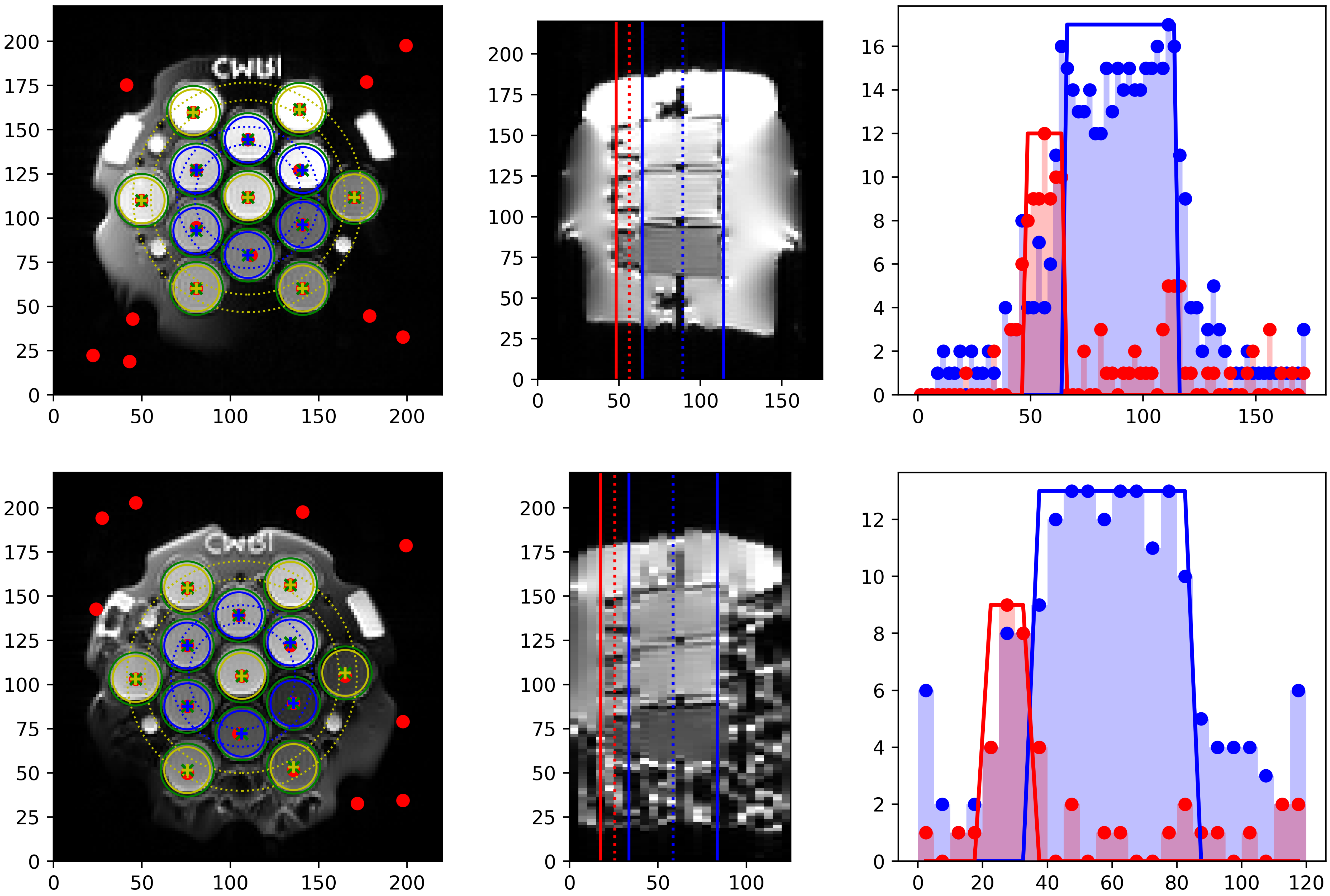

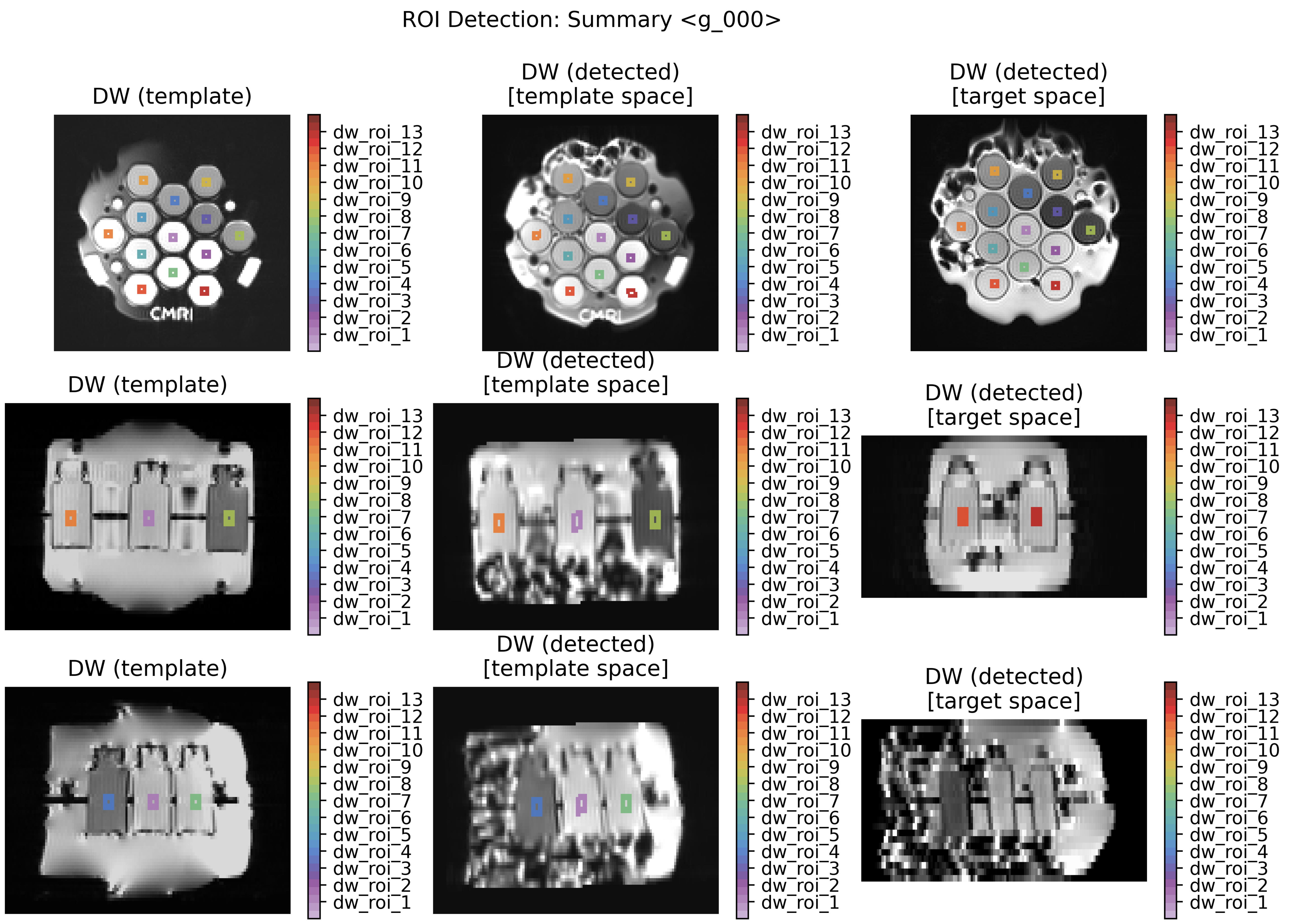
ROI Detection: Summary <g_000>
DW (template)
DW (detected) [template space]
DW (detected) [target space]
CMRI
dw_roi_13
dw_roi_12
dw_roi_11
dw_roi_10
dw_roi_9
dw_roi_8
dw_roi_7
dw_roi_6
dw_roi_5
dw_roi_4
dw_roi_3
dw_roi_2
dw_roi_1

ROI Detection: Summary <g_001>

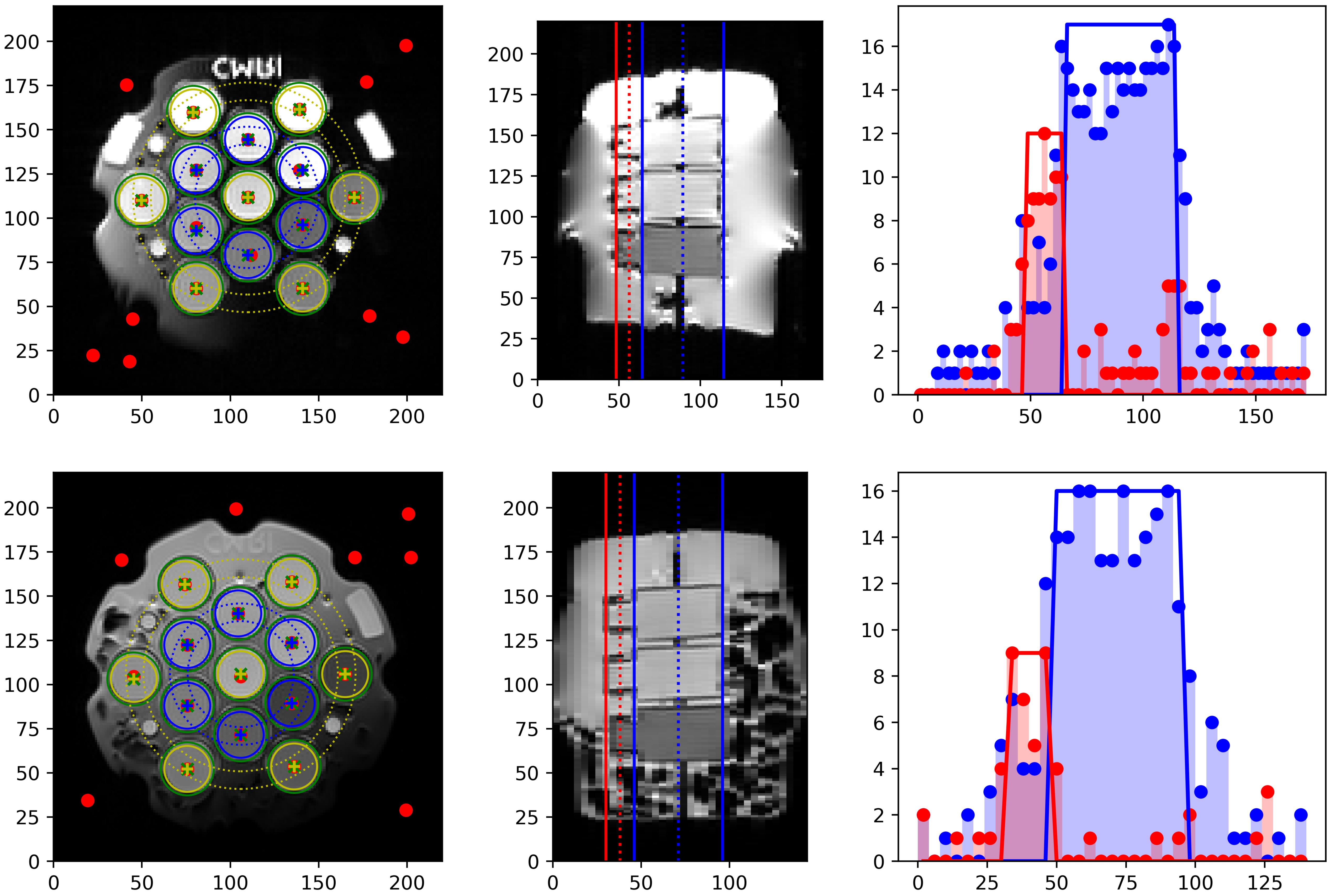

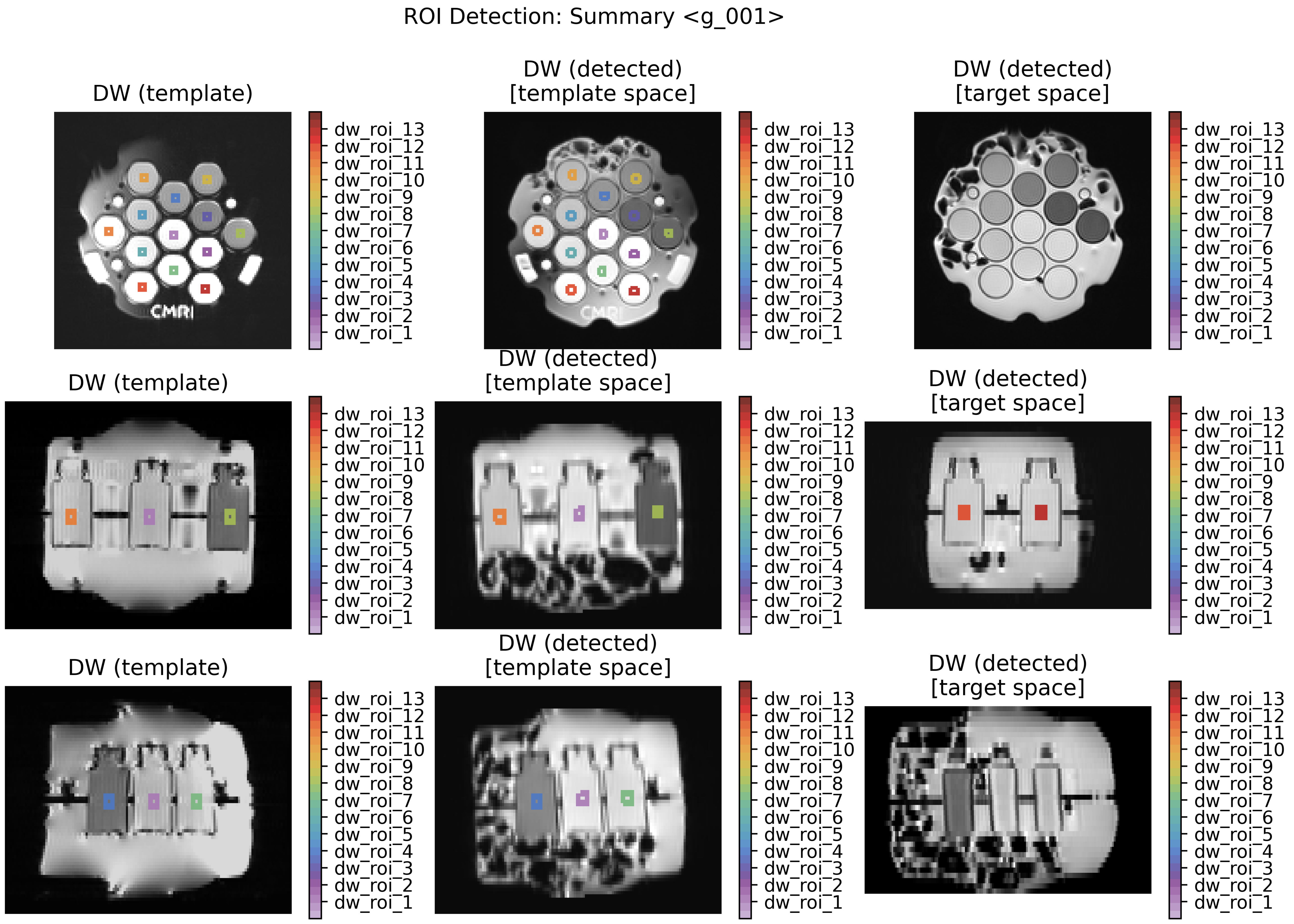
ROI Detection: Summary <g_001>
DW (template)
DW (detected)
[template space]
DW (detected)
[target space]
dw_roi_13
dw_roi_12
dw_roi_11
dw_roi_10
dw_roi_9
dw_roi_8
dw_roi_7
dw_roi_6
dw_roi_5
dw_roi_4
dw_roi_3
dw_roi_2
dw_roi_1
CMRI
DW (template)
DW (detected)
[template space]
DW (detected)
[target space]
DW (template)
DW (detected)
[template space]
DW (detected)
[target space]

CurveFit [DWCurveFit2param - AvROI_NrmROIMax] <dw_000>

ROI_DX	ROI LABEL	Sb_0	Sb_0_var	ADC	ADC_var	ADC_err	ADC_pct.err	ADC_ref	ADC_init	AVRGD	NORMLD	CLIPD
37	dw_roi_1	1.0	1.0	1123.4	1.1	14.4	1.3	1109.0	2000.0	True	True	False
38	dw_roi_2	1.0	1.0	1139.1	0.8	30.1	2.7	1109.0	2000.0	True	True	False
39	dw_roi_3	1.0	1.0	122.8	1.0	12.8	11.6	110.0	2000.0	True	True	False
40	dw_roi_4	1.0	1.0	230.5	1.9	10.5	4.8	220.0	2000.0	True	True	False
41	dw_roi_5	1.0	1.0	406.2	1.0	26.2	6.9	380.0	2000.0	True	True	False
42	dw_roi_6	1.0	1.0	606.0	1.5	27.0	4.7	579.0	2000.0	True	True	False
43	dw_roi_7	1.0	1.0	845.7	1.7	28.7	3.5	817.0	2000.0	True	True	False
44	dw_roi_8	1.0	1.0	134.1	4.5	24.1	22.0	110.0	2000.0	True	True	False
45	dw_roi_9	1.0	1.0	230.3	1.7	10.3	4.7	220.0	2000.0	True	True	False
46	dw_roi_10	1.0	1.0	418.4	1.4	38.4	10.1	380.0	2000.0	True	True	False
47	dw_roi_11	1.0	1.0	615.4	1.2	36.4	6.3	579.0	2000.0	True	True	False
48	dw_roi_12	1.0	1.0	866.0	2.1	49.0	6.0	817.0	2000.0	True	True	False
49	dw_roi_13	1.0	1.0	1184.7	2.9	75.7	6.8	1109.0	2000.0	True	True	False

SIGNAL EQUATION:

log(S(b)) = -b * ADC + log(Sb_0)

Parameter	Description	Init Val.	Min Val.	Max Val.
ADC	ADC	ADC	0.0	inf
Sb_0	Signal at b_0	max(S(b))	0.0	inf
b	b value	as measured	-	-

GOODNESS OF FIT:

ROI_DX	ROI LABEL	chisqr	redchi	aic	bic
37	dw_roi_1	nan	nan	nan	nan
38	dw_roi_2	nan	nan	nan	nan
39	dw_roi_3	nan	nan	nan	nan
40	dw_roi_4	nan	nan	nan	nan
41	dw_roi_5	nan	nan	nan	nan
42	dw_roi_6	nan	nan	nan	nan
43	dw_roi_7	nan	nan	nan	nan
44	dw_roi_8	nan	nan	nan	nan
45	dw_roi_9	nan	nan	nan	nan
46	dw_roi_10	nan	nan	nan	nan
47	dw_roi_11	nan	nan	nan	nan
48	dw_roi_12	nan	nan	nan	nan
49	dw_roi_13	nan	nan	nan	nan

chisqr : Chi-square statistic
redchi : Reduced Chi-square statistic
aic : Akaike Information Criterion statistic
bic : Bayesian Information Criterion statistic

CurveFit [DWCurveFit2param - AvROI_NrmROIMax] <dw_000>

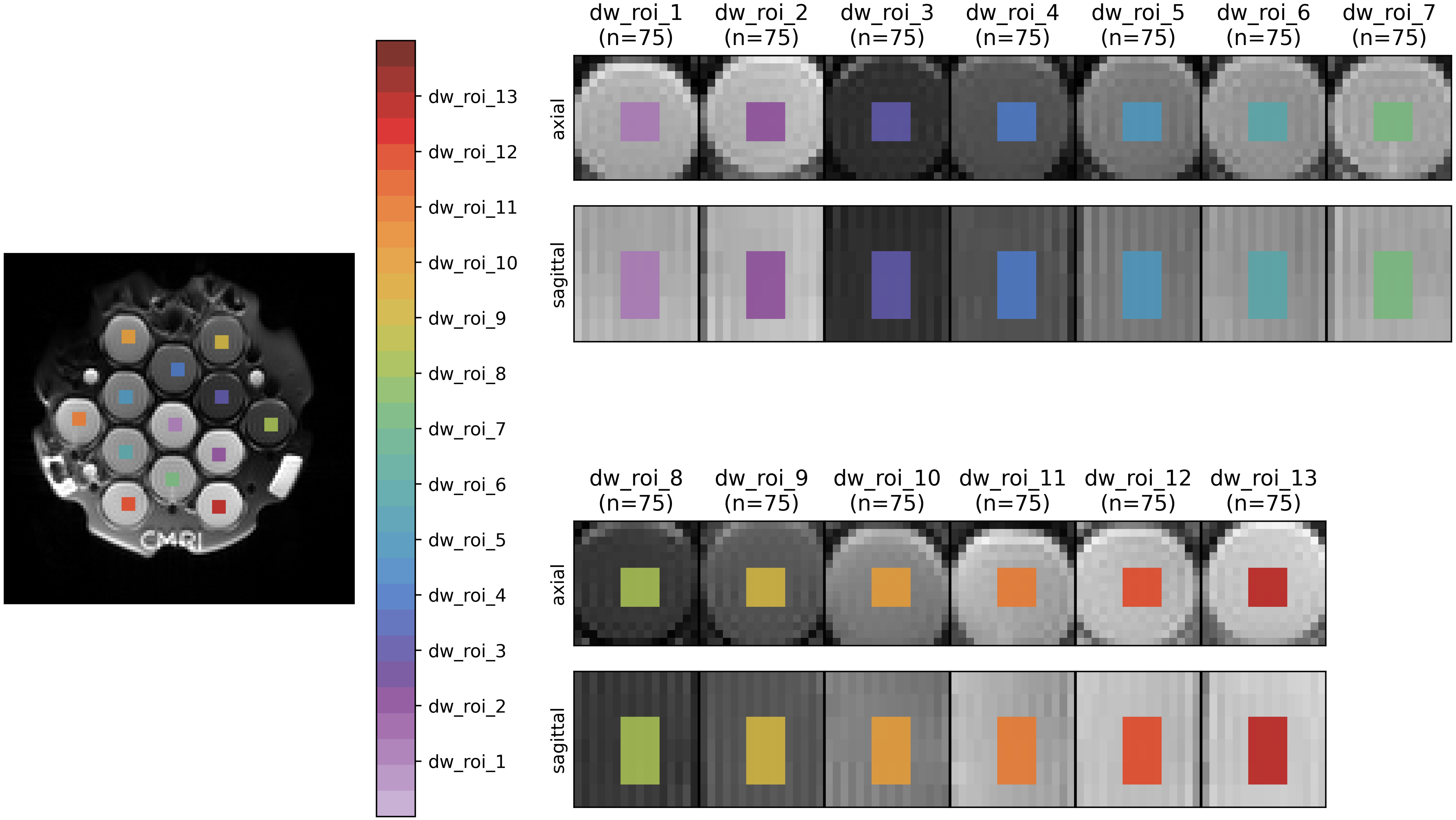

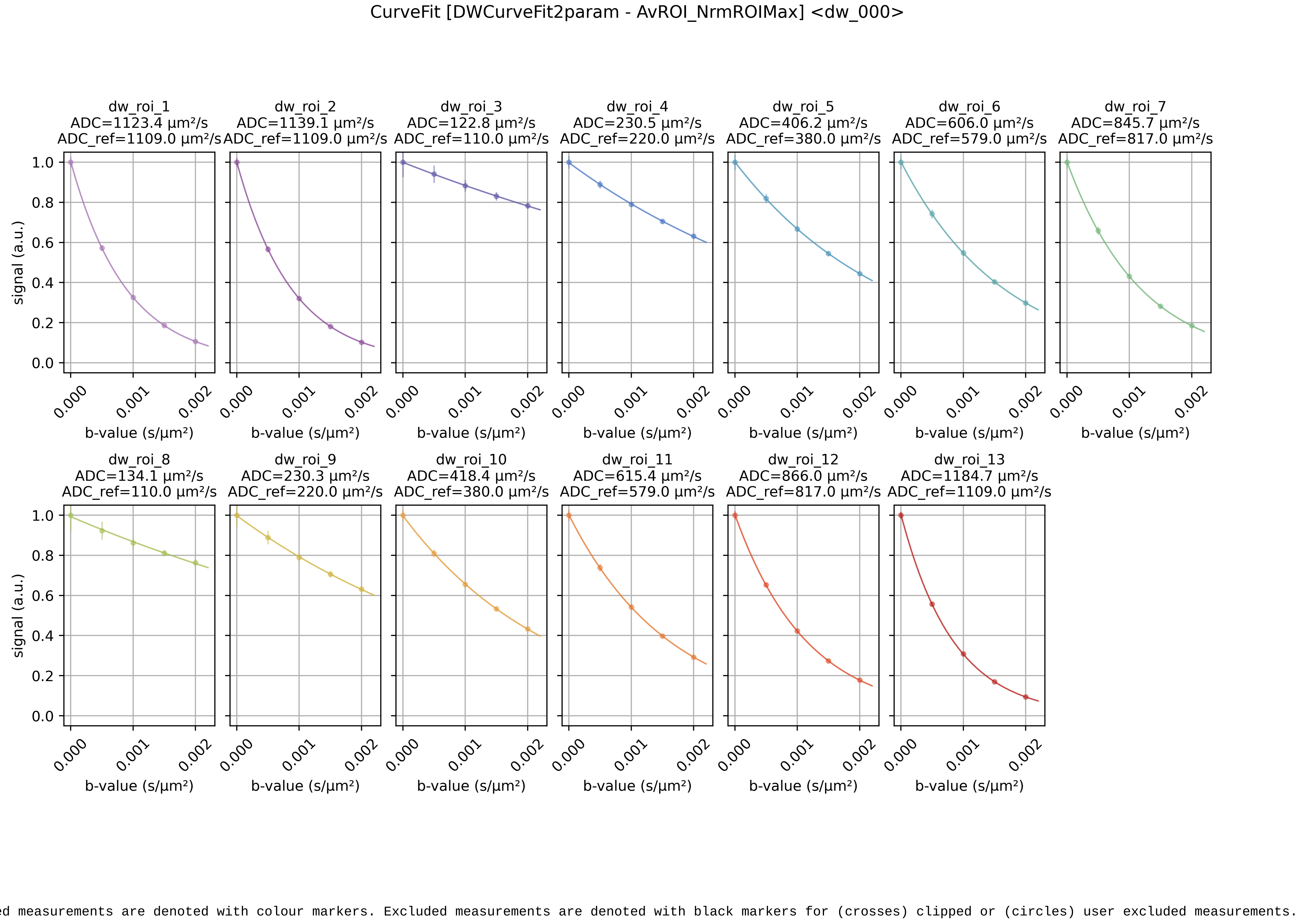
CurveFit [DWCurveFit2param - AvROI_NrmROIMax] <dw_000>
dw_roi_1
ADC=1123.4 µm²/s
ADC_ref=1109.0 µm²/s
dw_roi_2
ADC=1139.1 µm²/s
ADC_ref=1109.0 µm²/s
dw_roi_3
ADC=122.8 µm²/s
ADC_ref=110.0 µm²/s
dw_roi_4
ADC=230.5 µm²/s
ADC_ref=220.0 µm²/s
dw_roi_5
ADC=406.2 µm²/s
ADC_ref=380.0 µm²/s
dw_roi_6
ADC=606.0 µm²/s
ADC_ref=579.0 µm²/s
dw_roi_7
ADC=845.7 µm²/s
ADC_ref=817.0 µm²/s
dw_roi_8
ADC=134.1 µm²/s
ADC_ref=110.0 µm²/s
dw_roi_9
ADC=230.3 µm²/s
ADC_ref=220.0 µm²/s
dw_roi_10
ADC=418.4 µm²/s
ADC_ref=380.0 µm²/s
dw_roi_11
ADC=615.4 µm²/s
ADC_ref=579.0 µm²/s
dw_roi_12
ADC=866.0 µm²/s
ADC_ref=817.0 µm²/s
dw_roi_13
ADC=1184.7 µm²/s
ADC_ref=1109.0 µm²/s
signal (a.u.)
b-value (s/µm²)
0.0
0.2
0.4
0.6
0.8
1.0
0.000
0.001
0.002
Included measurements are denoted with colour markers. Excluded measurements are denoted with black markers for (crosses) clipped or (circles) user excluded measurements.

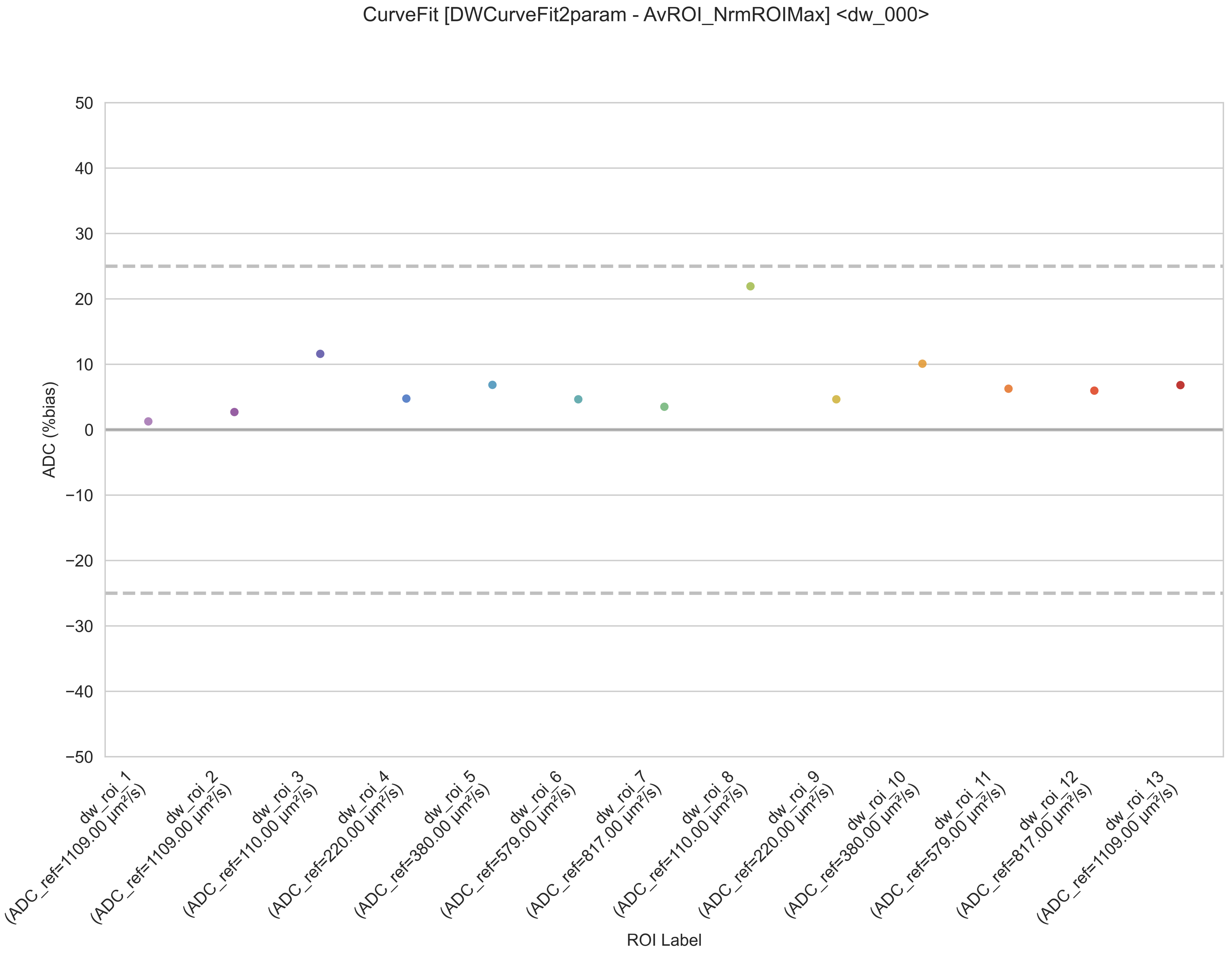

CurveFit [DWCurveFit2param - AvROI_NrmROIMax] <dw_000>
ADC (%bias)
50
40
30
20
10
0
−10
−20
−30
−40
−50
dw_roi_1 (ADC_ref=1109.00 µm²/s)
dw_roi_2 (ADC_ref=1109.00 µm²/s)
dw_roi_3 (ADC_ref=110.00 µm²/s)
dw_roi_4 (ADC_ref=220.00 µm²/s)
dw_roi_5 (ADC_ref=380.00 µm²/s)
dw_roi_6 (ADC_ref=579.00 µm²/s)
dw_roi_7 (ADC_ref=817.00 µm²/s)
dw_roi_8 (ADC_ref=110.00 µm²/s)
dw_roi_9 (ADC_ref=220.00 µm²/s)
dw_roi_10 (ADC_ref=380.00 µm²/s)
dw_roi_11 (ADC_ref=579.00 µm²/s)
dw_roi_12 (ADC_ref=817.00 µm²/s)
dw_roi_13 (ADC_ref=1109.00 µm²/s)
ROI Label

CurveFit [DWCurveFit2param - AvROI_NrmROIMax] <dw_001>

ROI_DX	ROI LABEL	Sb_0	Sb_0_var	ADC	ADC_var	ADC_err	ADC_pct.err	ADC_ref	ADC_init	AVRGD	NORMLD	CLIPD
37	dw_roi_1	1.0	1.0	1121.5	0.9	12.5	1.1	1109.0	2000.0	True	True	False
38	dw_roi_2	1.0	1.0	1139.6	0.6	30.6	2.8	1109.0	2000.0	True	True	False
39	dw_roi_3	1.0	1.0	121.1	1.4	11.1	10.0	110.0	2000.0	True	True	False
40	dw_roi_4	1.0	1.0	229.9	1.7	9.9	4.5	220.0	2000.0	True	True	False
41	dw_roi_5	1.0	1.0	404.9	1.2	24.9	6.6	380.0	2000.0	True	True	False
42	dw_roi_6	1.0	1.0	604.9	1.1	25.9	4.5	579.0	2000.0	True	True	False
43	dw_roi_7	1.0	1.0	845.9	1.7	28.9	3.5	817.0	2000.0	True	True	False
44	dw_roi_8	1.0	1.0	134.5	5.8	24.5	22.3	110.0	2000.0	True	True	False
45	dw_roi_9	1.0	1.0	231.1	2.3	11.1	5.0	220.0	2000.0	True	True	False
46	dw_roi_10	1.0	1.0	418.7	1.0	38.7	10.2	380.0	2000.0	True	True	False
47	dw_roi_11	1.0	1.0	614.9	1.2	35.9	6.2	579.0	2000.0	True	True	False
48	dw_roi_12	1.0	1.0	872.8	1.9	55.8	6.8	817.0	2000.0	True	True	False
49	dw_roi_13	1.0	1.0	1192.6	2.4	83.6	7.5	1109.0	2000.0	True	True	False

SIGNAL EQUATION:

log(S(b)) = -b * ADC + log(Sb_0)

Parameter	Description	Init Val.	Min Val.	Max Val.
ADC	ADC	ADC	0.0	inf
Sb_0	Signal at b_0	max(S(b))	0.0	inf
b	b value	as measured	-	-

GOODNESS OF FIT:

ROI_DX	ROI LABEL	chisqr	redchi	aic	bic
37	dw_roi_1	nan	nan	nan	nan
38	dw_roi_2	nan	nan	nan	nan
39	dw_roi_3	nan	nan	nan	nan
40	dw_roi_4	nan	nan	nan	nan
41	dw_roi_5	nan	nan	nan	nan
42	dw_roi_6	nan	nan	nan	nan
43	dw_roi_7	nan	nan	nan	nan
44	dw_roi_8	nan	nan	nan	nan
45	dw_roi_9	nan	nan	nan	nan
46	dw_roi_10	nan	nan	nan	nan
47	dw_roi_11	nan	nan	nan	nan
48	dw_roi_12	nan	nan	nan	nan
49	dw_roi_13	nan	nan	nan	nan

chisqr : Chi-square statistic
redchi : Reduced Chi-square statistic
aic : Akaike Information Criterion statistic
bic : Bayesian Information Criterion statistic

CurveFit [DWCurveFit2param - AvROI_NrmROIMax] <dw_001>

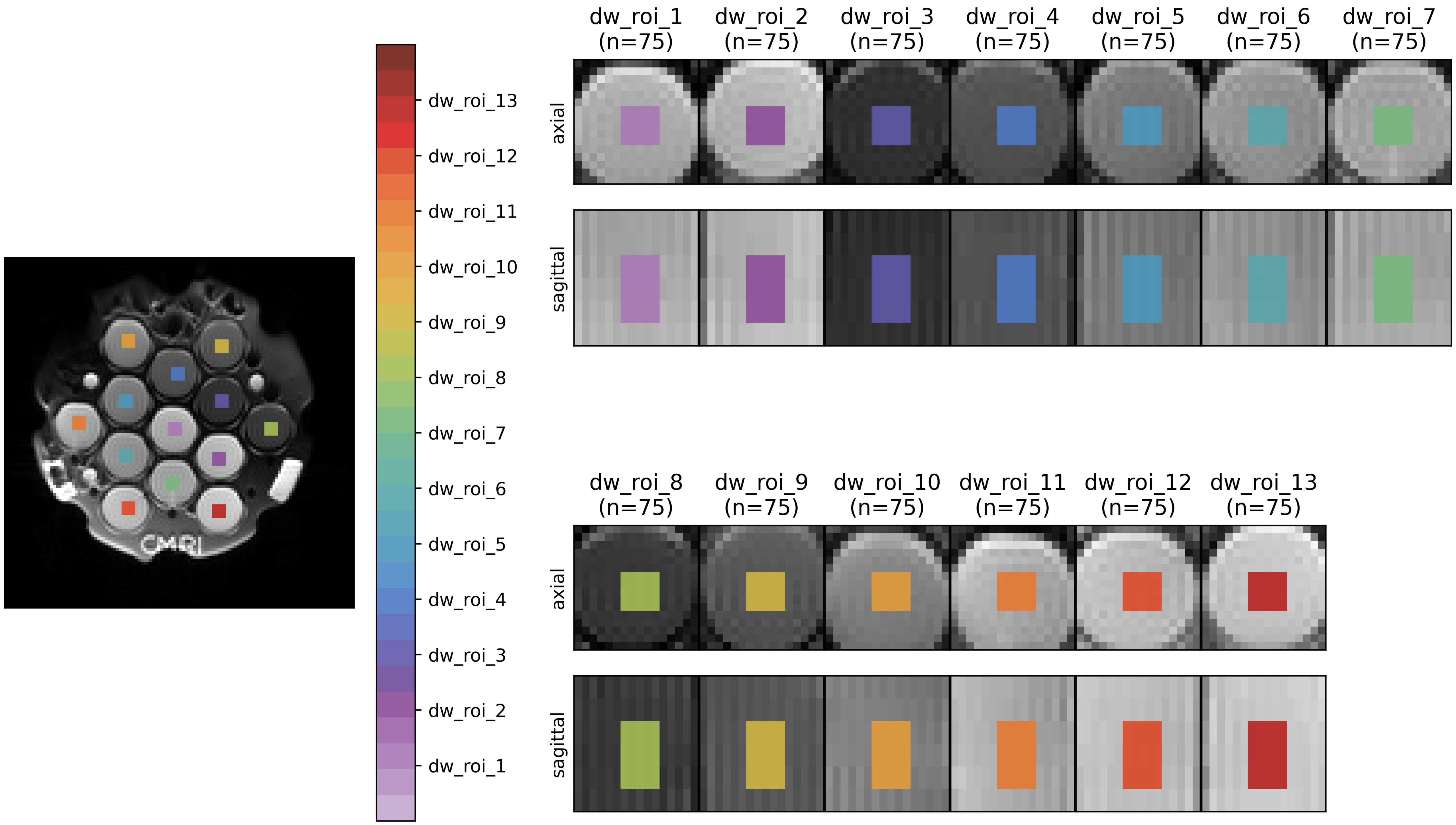

CurveFit [DWCurveFit2param - AvROI_NrmROIMax] <dw_001>

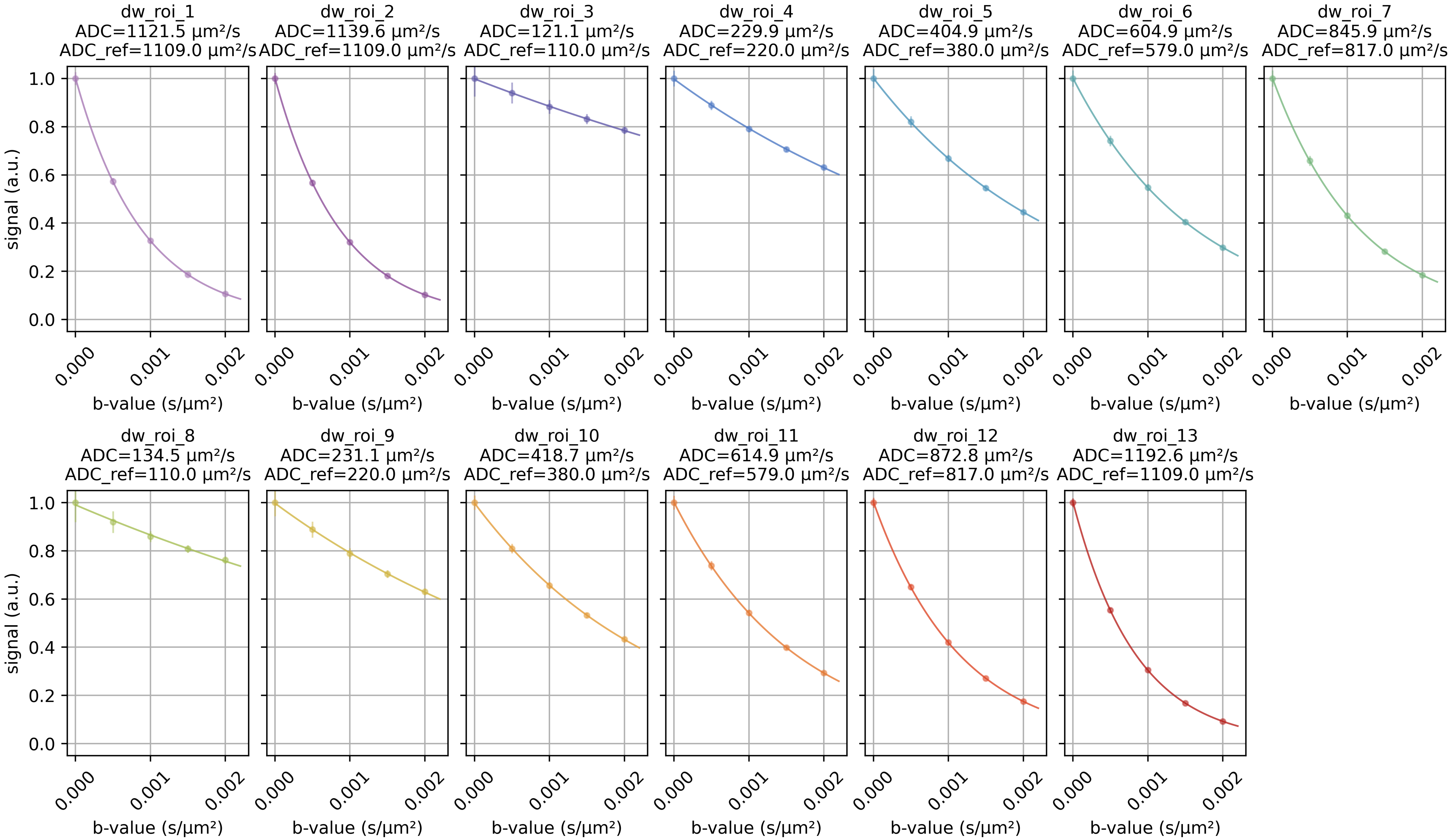

Included measurements are denoted with colour markers. Excluded measurements are denoted with black markers for (crosses) clipped or (circles) user excluded measurements.

CurveFit [DWCurveFit2param - AvROI_NrmROIMax] <dw_001>

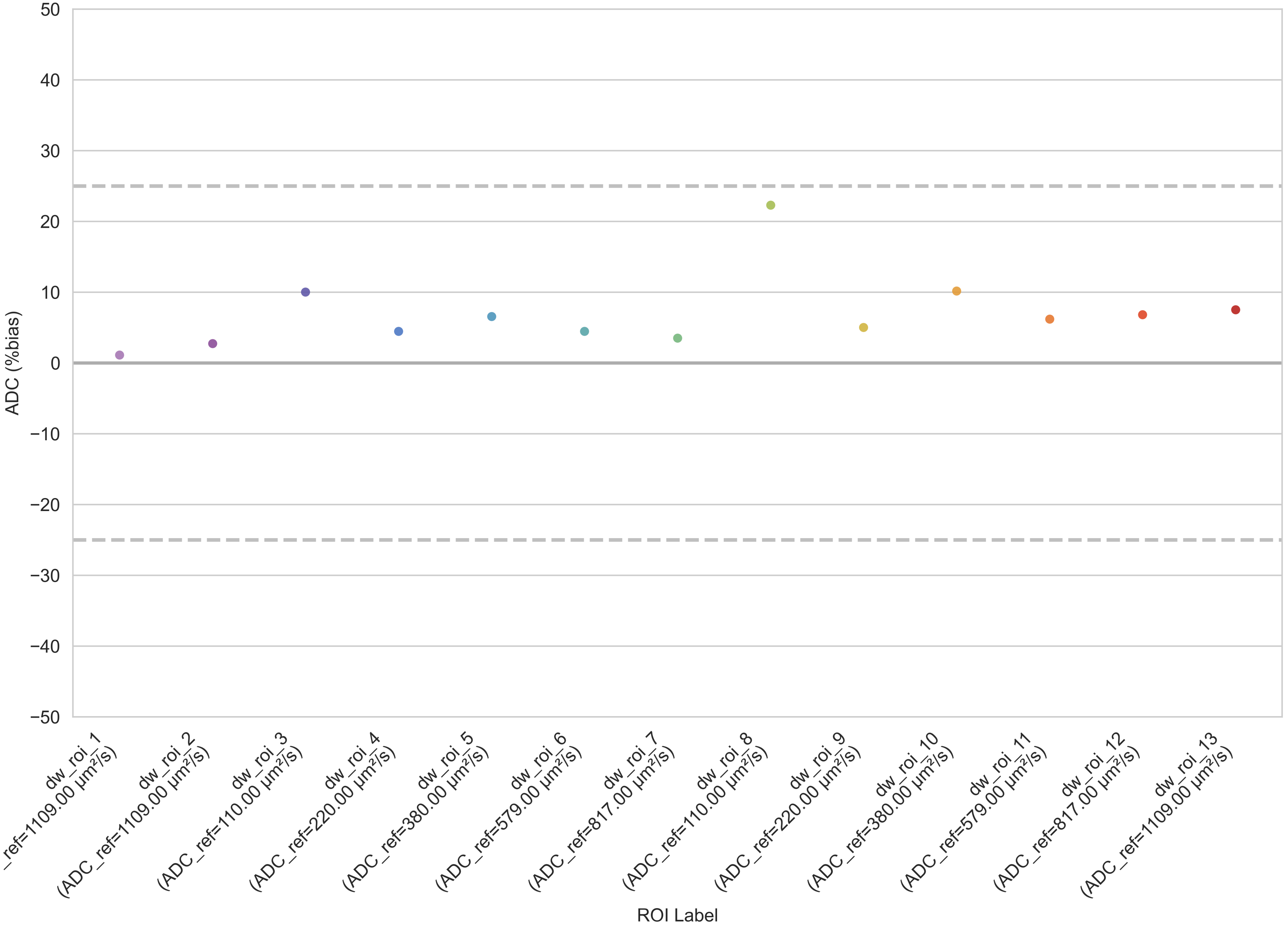

CurveFit [DWCurveFit2param - AvROI_NrmROIMax] <dw_002>

ROI_DX	ROI LABEL	Sb_0	Sb_0_var	ADC	ADC_var	ADC_err	ADC_pct.err	ADC_ref	ADC_init	AVRGD	NORMLD	CLIPD
37	dw_roi_1	1.0	1.0	1123.4	1.3	14.4	1.3	1109.0	2000.0	True	True	False
38	dw_roi_2	1.0	1.0	1140.8	0.7	31.8	2.9	1109.0	2000.0	True	True	False
39	dw_roi_3	1.0	1.0	120.9	1.7	10.9	9.9	110.0	2000.0	True	True	False
40	dw_roi_4	1.0	1.0	229.2	2.1	9.2	4.2	220.0	2000.0	True	True	False
41	dw_roi_5	1.0	1.0	405.1	0.8	25.1	6.6	380.0	2000.0	True	True	False
42	dw_roi_6	1.0	1.0	606.5	1.1	27.5	4.7	579.0	2000.0	True	True	False
43	dw_roi_7	1.0	1.0	849.3	1.4	32.3	3.9	817.0	2000.0	True	True	False
44	dw_roi_8	1.0	1.0	135.4	5.8	25.4	23.1	110.0	2000.0	True	True	False
45	dw_roi_9	1.0	1.0	229.9	2.3	9.9	4.5	220.0	2000.0	True	True	False
46	dw_roi_10	1.0	1.0	418.5	1.0	38.5	10.1	380.0	2000.0	True	True	False
47	dw_roi_11	1.0	1.0	615.7	1.0	36.7	6.3	579.0	2000.0	True	True	False
48	dw_roi_12	1.0	1.0	878.7	1.7	61.7	7.5	817.0	2000.0	True	True	False
49	dw_roi_13	1.0	1.0	1200.2	2.1	91.2	8.2	1109.0	2000.0	True	True	False

SIGNAL EQUATION:

log(S(b)) = -b * ADC + log(Sb_0)

Parameter	Description	Init Val.	Min Val.	Max Val.
ADC	ADC	ADC	0.0	inf
Sb_0	Signal at b_0	max(S(b))	0.0	inf
b	b value	as measured	-	-

GOODNESS OF FIT:

ROI_DX	ROI LABEL	chisqr	redchi	aic	bic
37	dw_roi_1	nan	nan	nan	nan
38	dw_roi_2	nan	nan	nan	nan
39	dw_roi_3	nan	nan	nan	nan
40	dw_roi_4	nan	nan	nan	nan
41	dw_roi_5	nan	nan	nan	nan
42	dw_roi_6	nan	nan	nan	nan
43	dw_roi_7	nan	nan	nan	nan
44	dw_roi_8	nan	nan	nan	nan
45	dw_roi_9	nan	nan	nan	nan
46	dw_roi_10	nan	nan	nan	nan
47	dw_roi_11	nan	nan	nan	nan
48	dw_roi_12	nan	nan	nan	nan
49	dw_roi_13	nan	nan	nan	nan

chisqr : Chi-square statistic
redchi : Reduced Chi-square statistic
aic : Akaike Information Criterion statistic
bic : Bayesian Information Criterion statistic

CurveFit [DWCurveFit2param - AvROI_NrmROIMax] <dw_002>

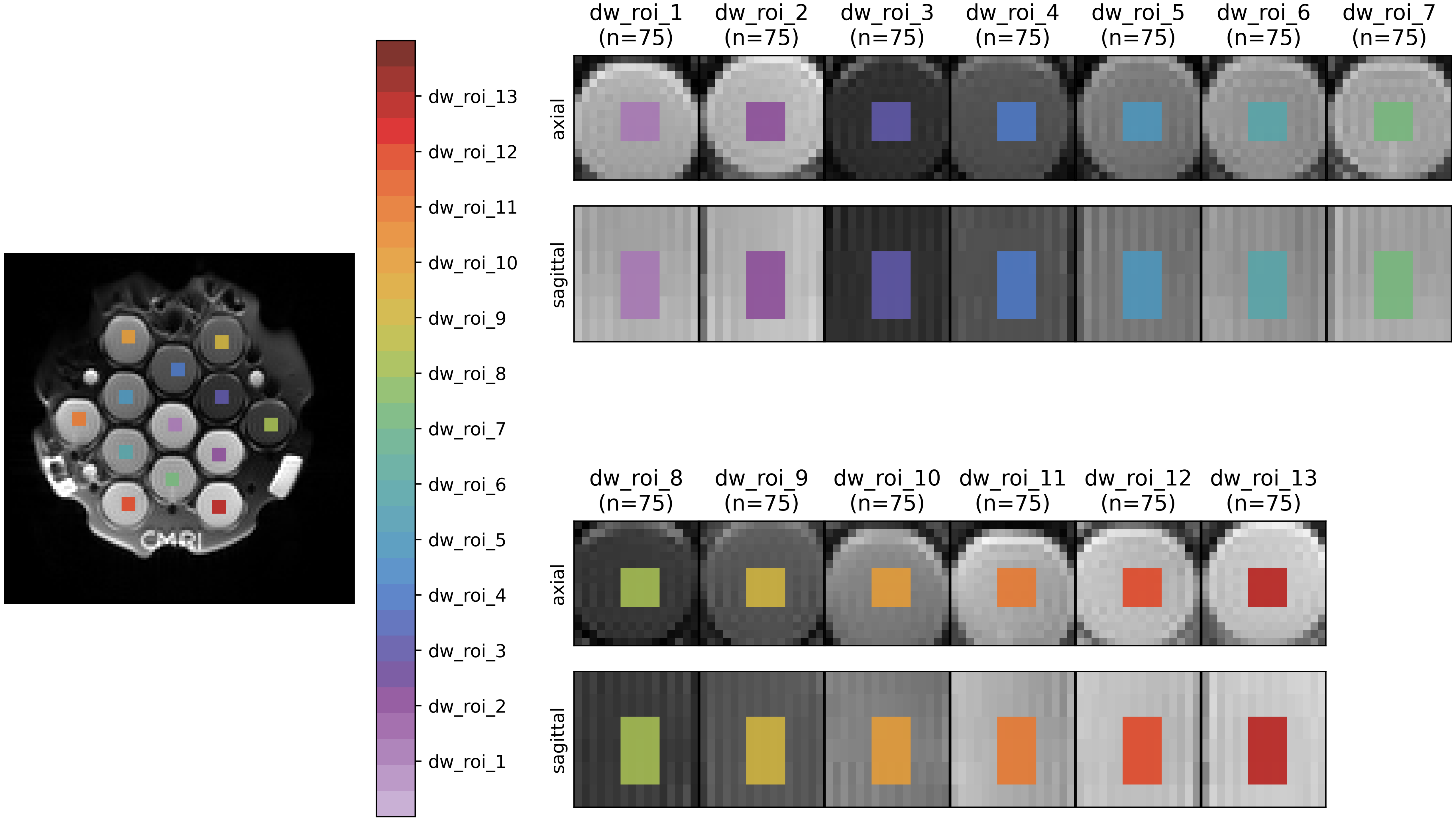

CurveFit [DWCurveFit2param - AvROI_NrmROIMax] <dw_002>

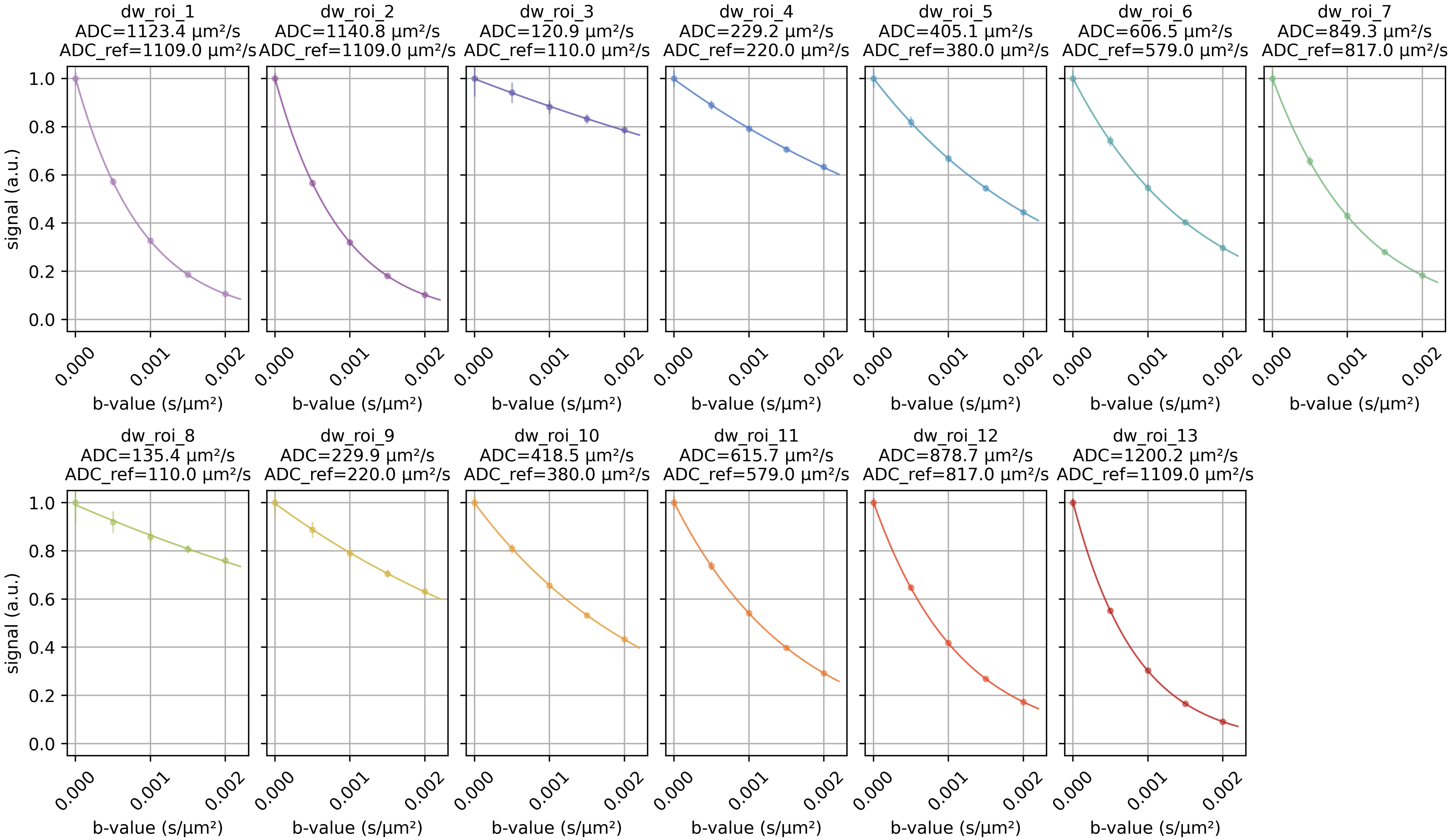

Included measurements are denoted with colour markers. Excluded measurements are denoted with black markers for (crosses) clipped or (circles) user excluded measurements.

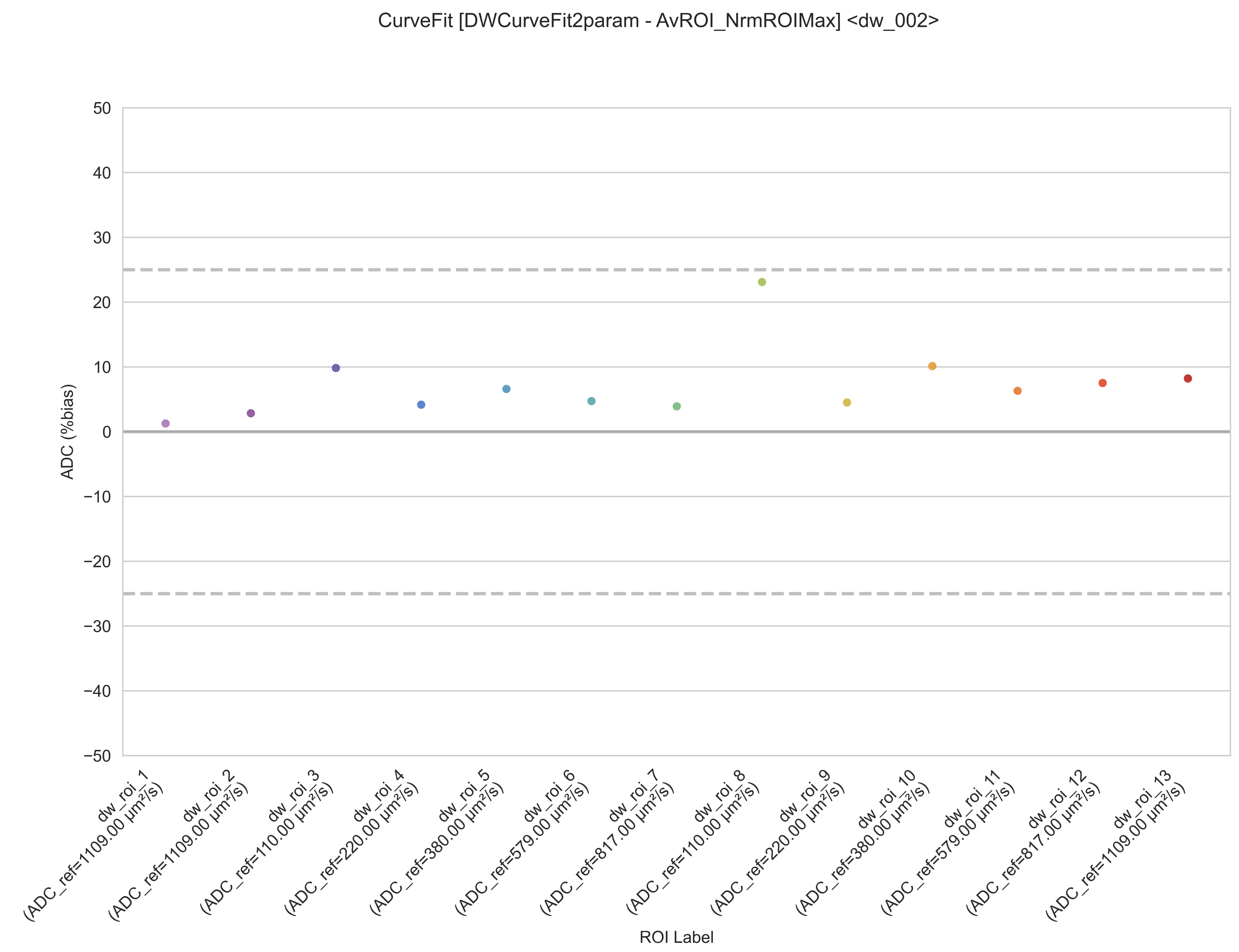
CurveFit [DWCurveFit2param - AvROI_NrmROIMax] <dw_002>
ADC (%bias)
50
40
30
20
10
0
−10
−20
−30
−40
−50
dw_roi_1 (ADC_ref=1109.00 µm²/s)
dw_roi_2 (ADC_ref=1109.00 µm²/s)
dw_roi_3 (ADC_ref=110.00 µm²/s)
dw_roi_4 (ADC_ref=220.00 µm²/s)
dw_roi_5 (ADC_ref=380.00 µm²/s)
dw_roi_6 (ADC_ref=579.00 µm²/s)
dw_roi_7 (ADC_ref=817.00 µm²/s)
dw_roi_8 (ADC_ref=110.00 µm²/s)
dw_roi_9 (ADC_ref=220.00 µm²/s)
dw_roi_10 (ADC_ref=380.00 µm²/s)
dw_roi_11 (ADC_ref=579.00 µm²/s)
dw_roi_12 (ADC_ref=817.00 µm²/s)
dw_roi_13 (ADC_ref=1109.00 µm²/s)
ROI Label

CurveFit [DWCurveFit2param - AvROI_NrmROIMax] <dw_003>

ROI_DX	ROI LABEL	Sb_0	Sb_0_var	ADC	ADC_var	ADC_err	ADC_pct.err	ADC_ref	ADC_init	AVRGD	NORMLD	CLIPD
37	dw_roi_1	1.0	1.0	1123.1	1.1	14.1	1.3	1109.0	2000.0	True	True	False
38	dw_roi_2	1.0	1.0	1143.5	1.0	34.5	3.1	1109.0	2000.0	True	True	False
39	dw_roi_3	1.0	1.0	123.5	2.4	13.5	12.3	110.0	2000.0	True	True	False
40	dw_roi_4	1.0	1.0	230.1	2.5	10.1	4.6	220.0	2000.0	True	True	False
41	dw_roi_5	1.0	1.0	405.1	1.0	25.1	6.6	380.0	2000.0	True	True	False
42	dw_roi_6	1.0	1.0	607.3	1.1	28.3	4.9	579.0	2000.0	True	True	False
43	dw_roi_7	1.0	1.0	851.2	1.4	34.2	4.2	817.0	2000.0	True	True	False
44	dw_roi_8	1.0	1.0	137.2	5.9	27.2	24.7	110.0	2000.0	True	True	False
45	dw_roi_9	1.0	1.0	231.4	2.5	11.4	5.2	220.0	2000.0	True	True	False
46	dw_roi_10	1.0	1.0	419.8	1.0	39.8	10.5	380.0	2000.0	True	True	False
47	dw_roi_11	1.0	1.0	616.5	1.1	37.5	6.5	579.0	2000.0	True	True	False
48	dw_roi_12	1.0	1.0	885.3	1.9	68.3	8.4	817.0	2000.0	True	True	False
49	dw_roi_13	1.0	1.0	1210.8	2.3	101.8	9.2	1109.0	2000.0	True	True	False

SIGNAL EQUATION:

log(S(b)) = -b * ADC + log(Sb_0)

Parameter	Description	Init Val.	Min Val.	Max Val.
ADC	ADC	ADC	0.0	inf
Sb_0	Signal at b_0	max(S(b))	0.0	inf
b	b value	as measured	-	-

GOODNESS OF FIT:

ROI_DX	ROI LABEL	chisqr	redchi	aic	bic
37	dw_roi_1	nan	nan	nan	nan
38	dw_roi_2	nan	nan	nan	nan
39	dw_roi_3	nan	nan	nan	nan
40	dw_roi_4	nan	nan	nan	nan
41	dw_roi_5	nan	nan	nan	nan
42	dw_roi_6	nan	nan	nan	nan
43	dw_roi_7	nan	nan	nan	nan
44	dw_roi_8	nan	nan	nan	nan
45	dw_roi_9	nan	nan	nan	nan
46	dw_roi_10	nan	nan	nan	nan
47	dw_roi_11	nan	nan	nan	nan
48	dw_roi_12	nan	nan	nan	nan
49	dw_roi_13	nan	nan	nan	nan

chisqr : Chi-square statistic
redchi : Reduced Chi-square statistic
aic : Akaike Information Criterion statistic
bic : Bayesian Information Criterion statistic

CurveFit [DWCurveFit2param - AvROI_NrmROIMax] <dw_003>

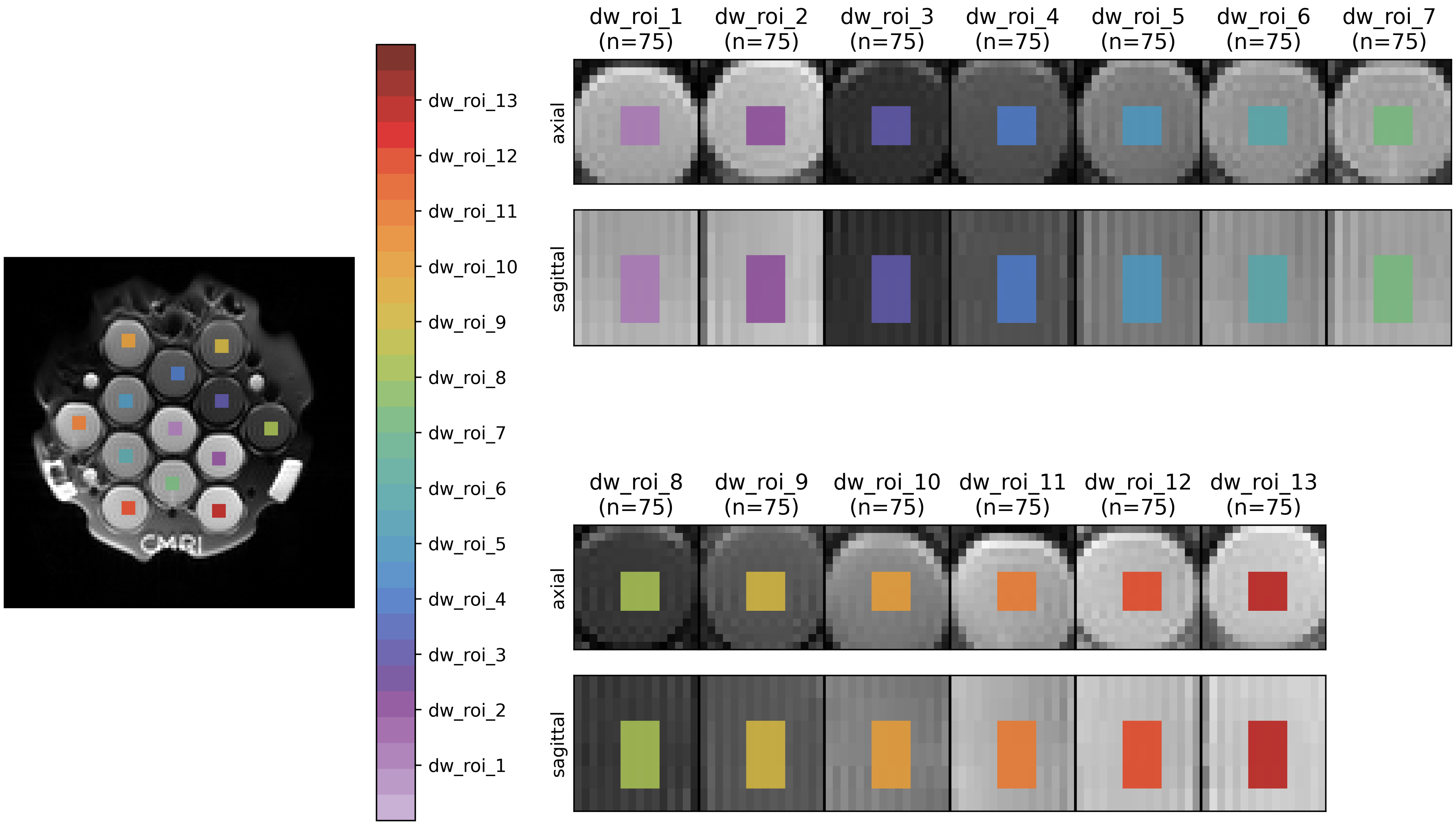

CurveFit [DWCurveFit2param - AvROI_NrmROIMax] <dw_003>

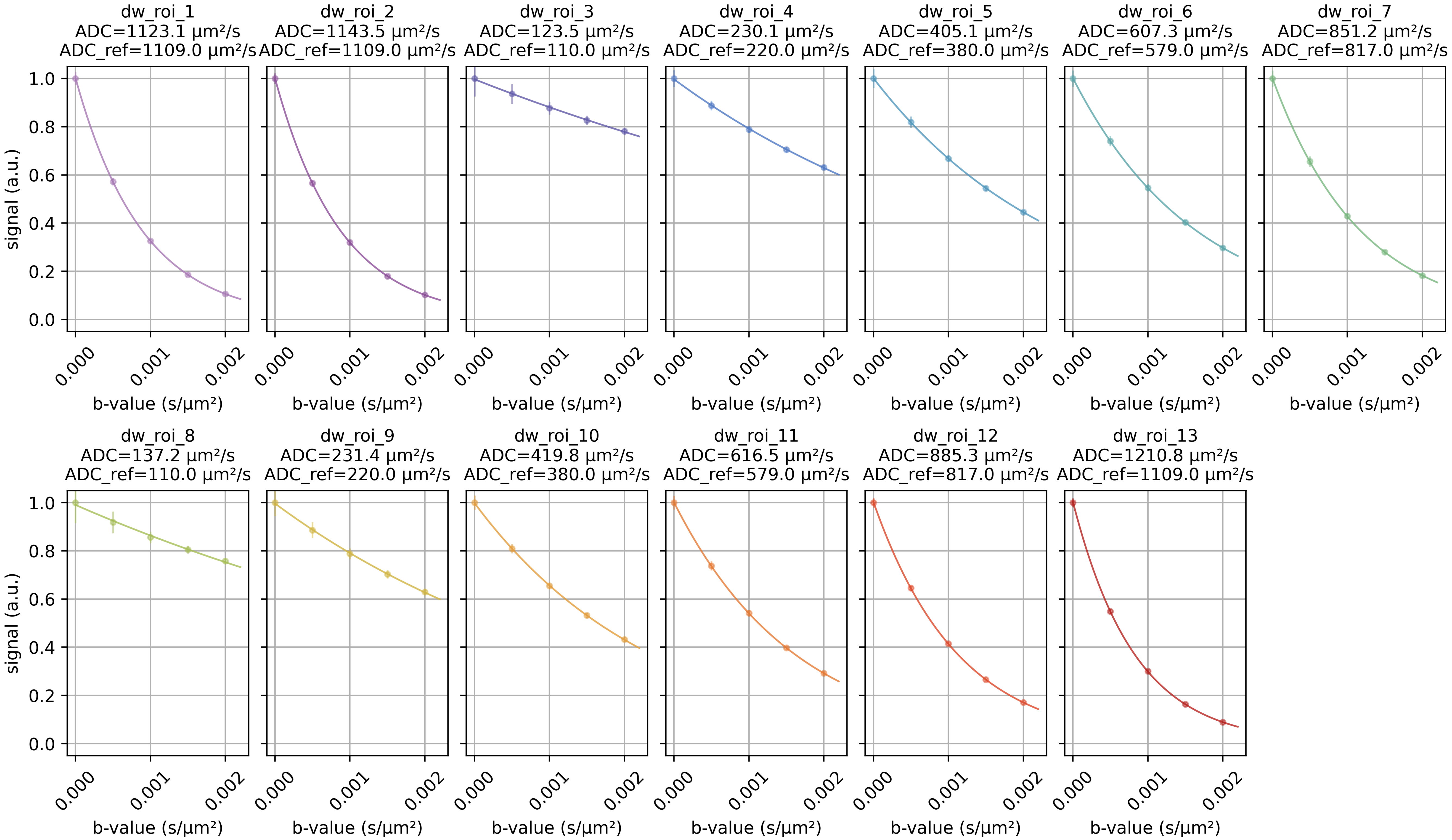

Included measurements are denoted with colour markers. Excluded measurements are denoted with black markers for (crosses) clipped or (circles) user excluded measurements.

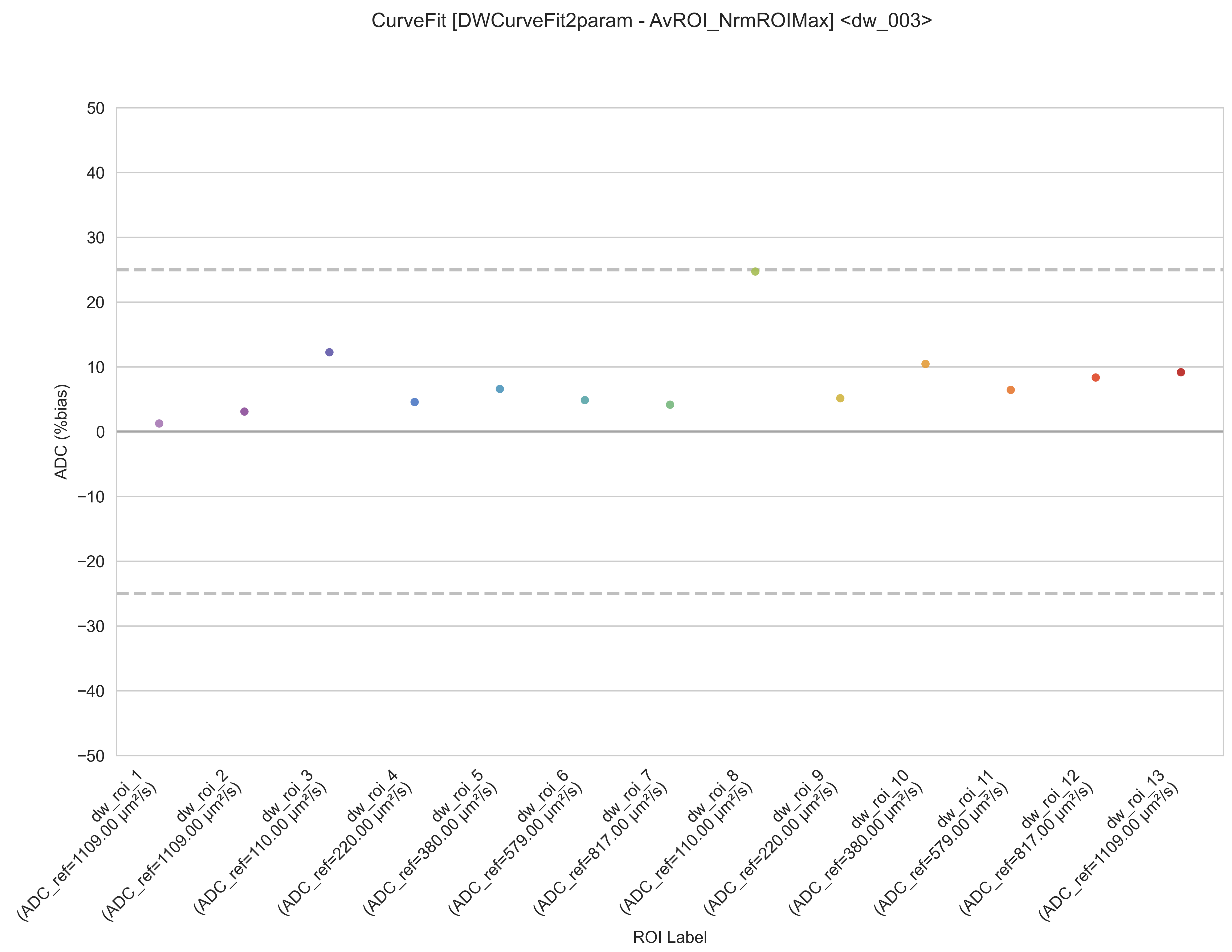
CurveFit [DWCurveFit2param - AvROI_NrmROIMax] <dw_003>
ADC (%bias)
50
40
30
20
10
0
−10
−20
−30
−40
−50
dw_roi_1 (ADC_ref=1109.00 µm²/s)
dw_roi_2 (ADC_ref=1109.00 µm²/s)
dw_roi_3 (ADC_ref=110.00 µm²/s)
dw_roi_4 (ADC_ref=220.00 µm²/s)
dw_roi_5 (ADC_ref=380.00 µm²/s)
dw_roi_6 (ADC_ref=579.00 µm²/s)
dw_roi_7 (ADC_ref=817.00 µm²/s)
dw_roi_8 (ADC_ref=110.00 µm²/s)
dw_roi_9 (ADC_ref=220.00 µm²/s)
dw_roi_10 (ADC_ref=380.00 µm²/s)
dw_roi_11 (ADC_ref=579.00 µm²/s)
dw_roi_12 (ADC_ref=817.00 µm²/s)
dw_roi_13 (ADC_ref=1109.00 µm²/s)
ROI Label

CurveFit [DWCurveFit2param - AvROI_NrmROIMax] <dw_004>

ROI_DX	ROI LABEL	Sb_0	Sb_0_var	ADC	ADC_var	ADC_err	ADC_pct.err	ADC_ref	ADC_init	AVRGD	NORMLD	CLIPD
37	dw_roi_1	1.0	1.0	1125.5	0.0	16.5	1.5	1109.0	2000.0	True	True	False
38	dw_roi_2	1.0	1.0	1140.0	0.0	31.0	2.8	1109.0	2000.0	True	True	False
39	dw_roi_3	1.0	1.0	115.5	0.0	5.5	5.0	110.0	2000.0	True	True	False
40	dw_roi_4	1.0	1.0	227.7	0.0	7.7	3.5	220.0	2000.0	True	True	False
41	dw_roi_5	1.0	1.0	413.0	0.0	33.0	8.7	380.0	2000.0	True	True	False
42	dw_roi_6	1.0	1.0	614.5	0.0	35.5	6.1	579.0	2000.0	True	True	False
43	dw_roi_7	1.0	1.0	859.7	0.0	42.7	5.2	817.0	2000.0	True	True	False
44	dw_roi_8	1.0	1.0	117.3	0.0	7.3	6.6	110.0	2000.0	True	True	False
45	dw_roi_9	1.0	1.0	226.1	0.0	6.1	2.8	220.0	2000.0	True	True	False
46	dw_roi_10	1.0	1.0	415.5	0.0	35.5	9.4	380.0	2000.0	True	True	False
47	dw_roi_11	1.0	1.0	618.7	0.0	39.7	6.9	579.0	2000.0	True	True	False
48	dw_roi_12	1.0	1.0	889.2	0.0	72.2	8.8	817.0	2000.0	True	True	False
49	dw_roi_13	1.0	1.0	1205.5	0.0	96.5	8.7	1109.0	2000.0	True	True	False

SIGNAL EQUATION:

log(S(b)) = -b * ADC + log(Sb_0)

Parameter	Description	Init Val.	Min Val.	Max Val.
ADC	ADC	ADC	0.0	inf
Sb_0	Signal at b_0	max(S(b))	0.0	inf
b	b value	as measured	-	-

GOODNESS OF FIT:

ROI_DX	ROI LABEL	chisqr	redchi	aic	bic
37	dw_roi_1	nan	nan	nan	nan
38	dw_roi_2	nan	nan	nan	nan
39	dw_roi_3	nan	nan	nan	nan
40	dw_roi_4	nan	nan	nan	nan
41	dw_roi_5	nan	nan	nan	nan
42	dw_roi_6	nan	nan	nan	nan
43	dw_roi_7	nan	nan	nan	nan
44	dw_roi_8	nan	nan	nan	nan
45	dw_roi_9	nan	nan	nan	nan
46	dw_roi_10	nan	nan	nan	nan
47	dw_roi_11	nan	nan	nan	nan
48	dw_roi_12	nan	nan	nan	nan
49	dw_roi_13	nan	nan	nan	nan

chisqr : Chi-square statistic
redchi : Reduced Chi-square statistic
aic : Akaike Information Criterion statistic
bic : Bayesian Information Criterion statistic

CurveFit [DWCurveFit2param - AvROI_NrmROIMax] <dw_004>

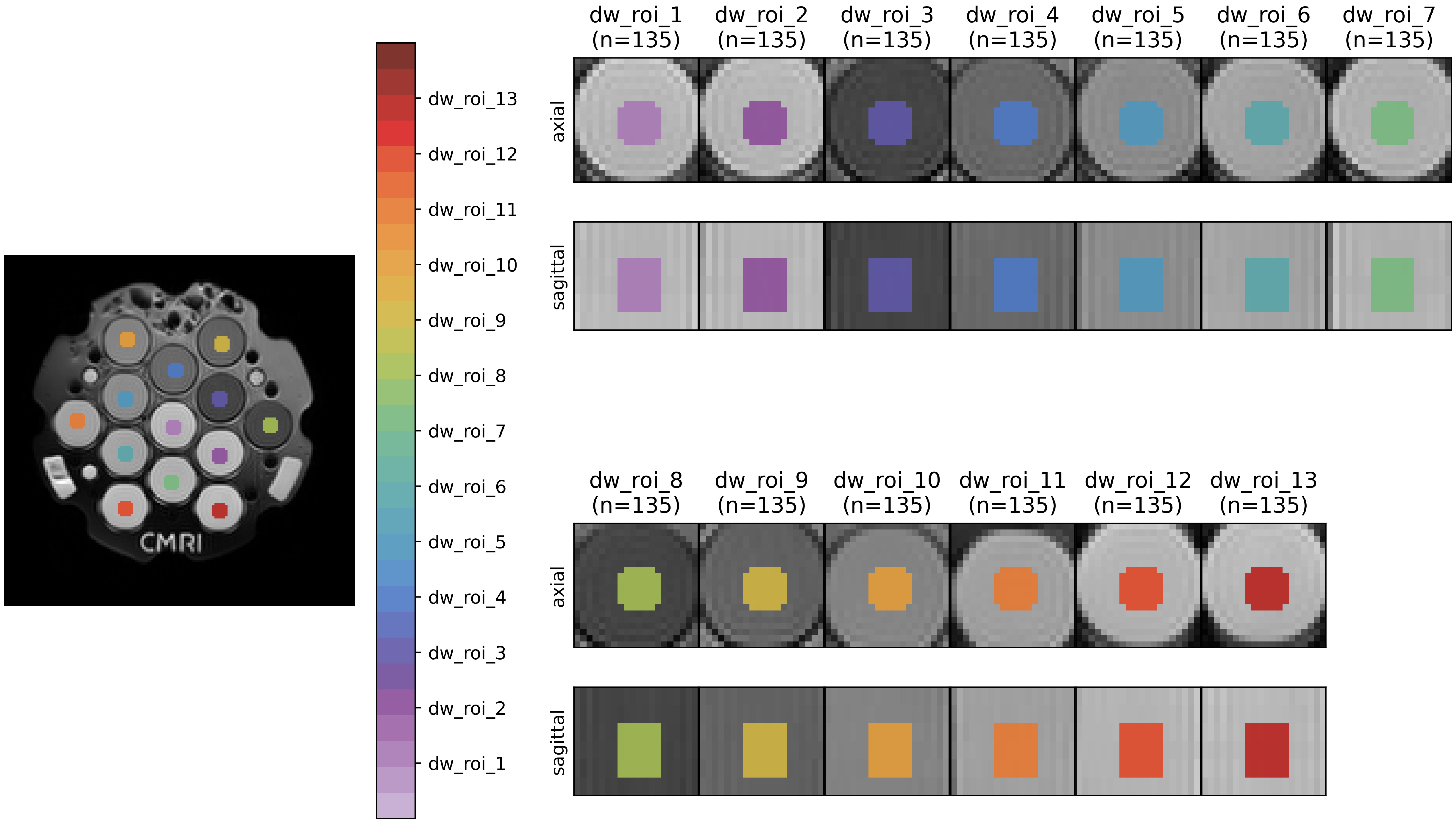

CurveFit [DWCurveFit2param - AvROI_NrmROIMax] <dw_004>

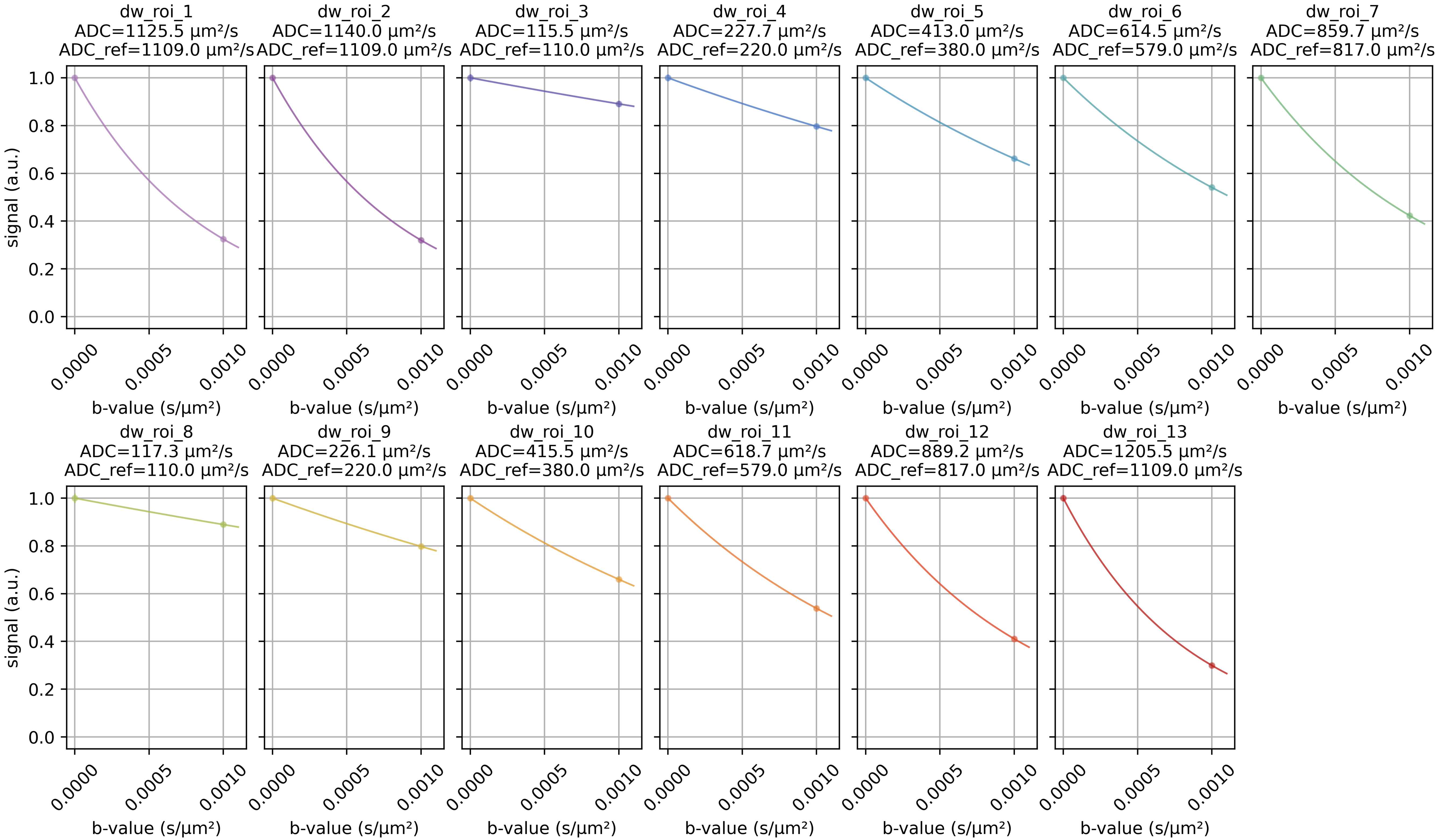

Included measurements are denoted with colour markers. Excluded measurements are denoted with black markers for (crosses) clipped or (circles) user excluded measurements.

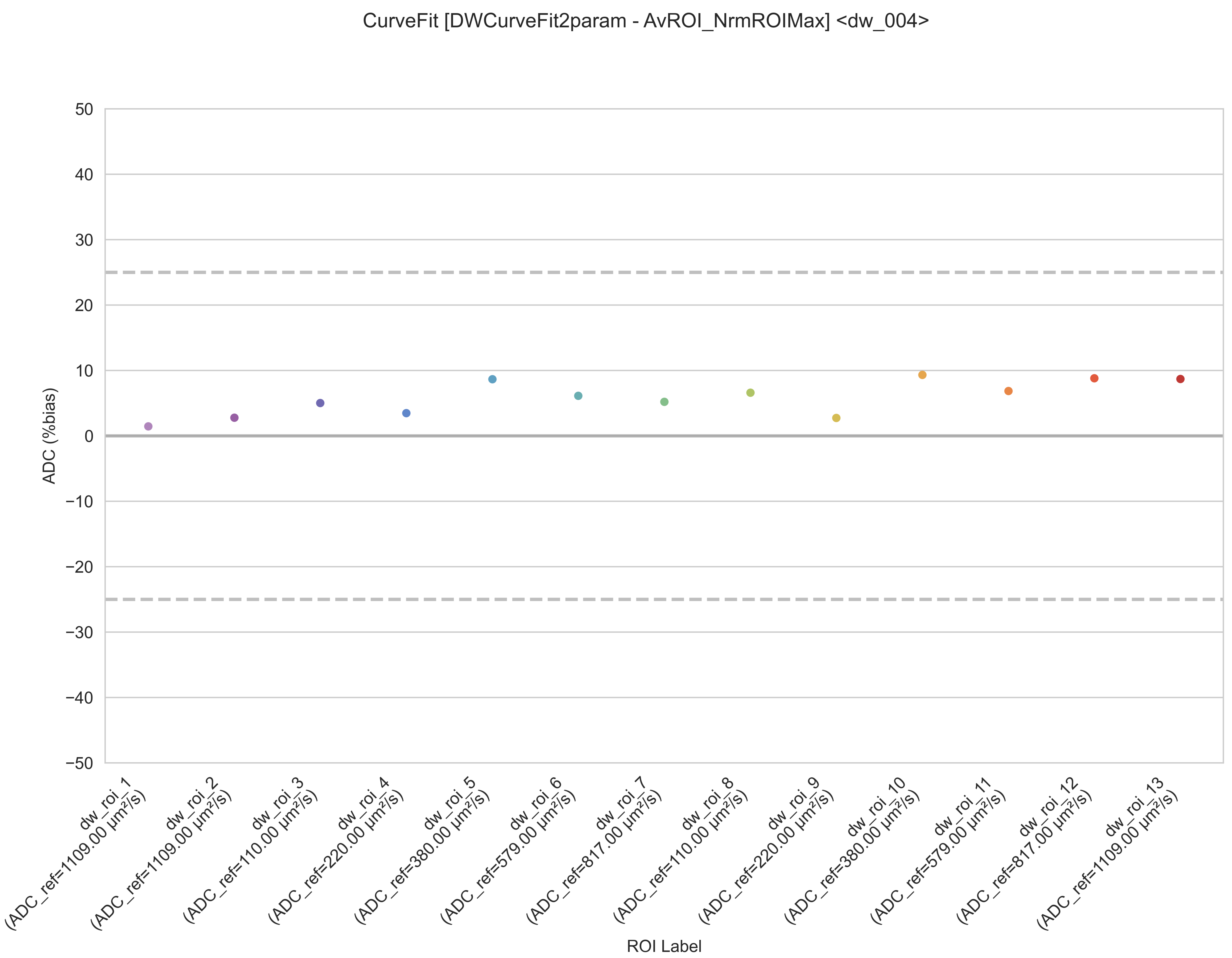
CurveFit [DWCurveFit2param - AvROI_NrmROIMax] <dw_004>
ADC (%bias)
50
40
30
20
10
0
−10
−20
−30
−40
−50
dw_roi_1 (ADC_ref=1109.00 µm²/s)
dw_roi_2 (ADC_ref=1109.00 µm²/s)
dw_roi_3 (ADC_ref=110.00 µm²/s)
dw_roi_4 (ADC_ref=220.00 µm²/s)
dw_roi_5 (ADC_ref=380.00 µm²/s)
dw_roi_6 (ADC_ref=579.00 µm²/s)
dw_roi_7 (ADC_ref=817.00 µm²/s)
dw_roi_8 (ADC_ref=110.00 µm²/s)
dw_roi_9 (ADC_ref=220.00 µm²/s)
dw_roi_10 (ADC_ref=380.00 µm²/s)
dw_roi_11 (ADC_ref=579.00 µm²/s)
dw_roi_12 (ADC_ref=817.00 µm²/s)
dw_roi_13 (ADC_ref=1109.00 µm²/s)
ROI Label

Repeatability metrics across 4 short-term scan repeats:

- CurveFit [DWCurveFit2param - AvROI_NrmROIMax] <dw_000>
- CurveFit [DWCurveFit2param - AvROI_NrmROIMax] <dw_001>
- CurveFit [DWCurveFit2param - AvROI_NrmROIMax] <dw_002>
- CurveFit [DWCurveFit2param - AvROI_NrmROIMax] <dw_003>
- CurveFit [DWCurveFit2param - AvROI_NrmROIMax] <dw_004>

ROI LABEL	ref	mean	std	RC_st	CV_st	bias (%)
dw_roi_1	1109.00	1123.40	1.42	3.94	0.13	1.30
dw_roi_2	1109.00	1140.61	1.73	4.80	0.15	2.85
dw_roi_3	110.00	120.74	3.12	8.65	2.59	9.76
dw_roi_4	220.00	229.47	1.12	3.10	0.49	4.31
dw_roi_5	380.00	406.87	3.45	9.55	0.85	7.07
dw_roi_6	579.00	607.85	3.83	10.61	0.63	4.98
dw_roi_7	817.00	850.36	5.71	15.81	0.67	4.08
dw_roi_8	110.00	131.72	8.17	22.63	6.20	19.75
dw_roi_9	220.00	229.75	2.15	5.94	0.93	4.43
dw_roi_10	380.00	418.20	1.59	4.40	0.38	10.05
dw_roi_11	579.00	616.26	1.50	4.15	0.24	6.44
dw_roi_12	817.00	878.40	9.36	25.91	1.07	7.51
dw_roi_13	1109.00	1198.74	10.32	28.60	0.86	8.09

SUMMARY METRICS:

Metric	Description	Symbol
ref	Reference ADC value as measured by NIST	ADC_ref
mean	Mean ADC value across 4 short-term repeats	ADC_mean
std	Standard deviation of ADC values across 4 short-term repeats	SD
RC_st	Repeatability coefficient	RC_st = 2.77 * SD
CV_st	Coefficient of variation	CV_st = 100% * (SD/ADC_mean)
bias (%)	%Bias	bias(%) = 100% * (ADC_mean - ADC_ref)/ADC_ref
